# Paired Electrosynthesis at Interdigitated Microband Electrodes: Exploring Diffusion and Reaction Zones in the Absence of a Supporting Electrolyte

**DOI:** 10.1021/acsmeasuresciau.4c00009

**Published:** 2024-04-17

**Authors:** Tingran Liu, Evaldo Batista Carneiro-Neto, Ernesto Pereira, James E. Taylor, Philip J. Fletcher, Frank Marken

**Affiliations:** † Department of Chemistry, University of Bath, Claverton Down, Bath BA2 7AY, U.K.; ‡ Department of Chemistry, Federal University of São Carlos, Rod. Washington Luiz, Km 235, CEP 13565-905 São Carlos, SP, Brazil; § Materials & Chemical Characterisation Facility, MC^2^, University of Bath, Bath BA2 7AY, U.K.

**Keywords:** voltammetry, microreactor, paired electrosynthesis, finite elements, electro-hydrogenation, sustainable chemistry

## Abstract

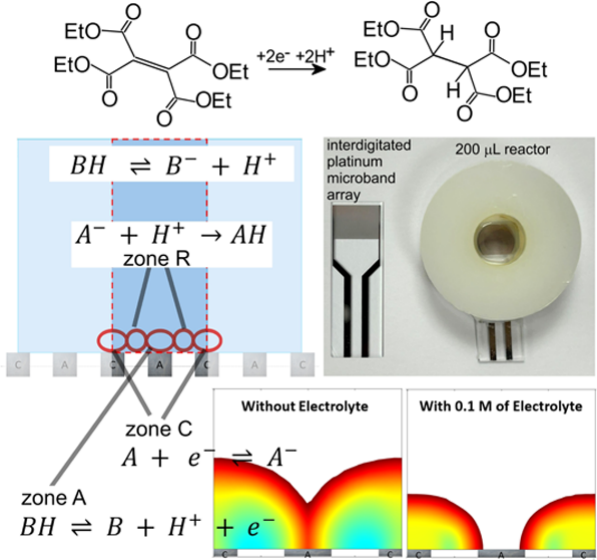

Electrosynthesis traditionally requires dedicated reactor systems and an added electrolyte, although some paired electrosynthesis processes are possible at interdigitated microband electrodes simply immersed in solution and without an intentionally added electrolyte. Here, 1,1′-ferrocenedimethanol oxidation and activated olefin electro-hydrogenation reactions are investigated as model processes at a Pt–Pt interdigitated microband array electrode with 5 μm width and with 5 μm interelectrode gap. Voltammetric responses for electro-hydrogenation are discussed, and product yields are determined in methanol (MeOH) in the presence/absence of an added electrolyte (LiClO_4_). An isotope effect is observed in CH_3_OD solvent, leading to olefin monodeuteration linked to a fast EC-type process close to the cathode surface (in the cathode reaction zone) rather than to charge annihilation in the interelectrode zone. A finite element simulation is employed to visualize/discuss reaction zones and to contrast the rate of charge annihilation processes with/without a supporting electrolyte.

## 1 Introduction

The search for new methodologies in electrosynthesis^1,2^ is linked to the desire to transform many aspects to chemical synthesis away from using reagents (which produce waste) to redox steps driven by electron transfer and by sustainable electricity.^3^ In particular, in the biorefinery and biofuel contexts, paired redox transformations could be valuable.^4^ New methodologies have been proposed based on microfluidic reactors,^5^ the use of driven fuel cell reactors,^6^ multiphase reactors,^7^ ultrasound^8^ or microwave/radio frequency-activated^9^ processes, and paired electrosynthesis,^10^ which takes into account the coupled nature of electrochemical processes linking oxidation and reduction into a single (more complex) paired electrode process.

Similar to light-driven photochemical or photocatalytic processes in organic synthesis,^11^ paired electricity-driven electrosynthetic processes are possible due to close spatial vicinity of the anode and the cathode to couple reactions associated with oxidation and with reduction, respectively. In a recent pioneering work by Mo, Jensen, and co-workers,^12^ the link from photochemical synthesis (producing locally oxidation and reduction events due to excitation with a photon) to paired electrosynthesis at interdigitated microband arrays (oxidation and reduction in close vicinity as defined by the interelectrode gap) has been pointed out. Zahrt and Jensen suggested that paired electrosynthetic processes at interdigitated microband arrays can be employed in the systematic exploration/discovery of reaction spaces in conjunction with machine learning algorithms.^13^ Paired electrosyntheses at interdigitated microband arrays are associated with considerable mechanistic complexity, and in addition to reaction spaces, new tools/theories need to be developed to allow better prediction of reaction zones, pathways, rates, and yields.

Girault and co-workers introduced platinum interdigitated band electrodes into electrosynthesis^14^ and demonstrated microfluidic reactors for furan methoxylation,^15,16^ for hypochlorite/peroxide production,^17^ and for epoxidation reactions.^18^ Computational analysis of reactions at interdigitated band arrays under flow was reported.^19^ Apart from this pioneering work, interdigitated microarray electrodes have been developed mainly for sensing^20,21^ and implemented with electrode materials such as platinum,^22^ carbon,^23^ or boron-doped diamond.^24^ Interdigitated nanoband arrays have been reported down to 100 nm electrode width.^25^ The width between electrodes is associated with the interelectrode diffusion time, which can be estimated as τ = δ^2^/(π*D*) = 8 ms for a gap of 5 μm and a typical diffusion coefficient of 10^–9^ m^2^ s^–1^. Any reactions involving products from both anode and cathode processes are limited by the interelectrode diffusion time. This is limited by the geometry. However, it is shown here that additional migration effects in the absence of an intentionally added electrolyte can speed up interelectrode transport (*vide infra*).

In paired electrosynthesis,^26−28^ anode and cathode can be spaced with centimeter gaps in a single compartment^29^ or located much more closely together (micrometers).^30^ With a large interelectrode gap, the supporting electrolyte is essential to maintain some degree of electrical conductivity and to limit energy losses. However, with closely spaced electrodes, the requirement for the supporting electrolyte can be dropped in part due to localized formation of ionic species (in self-supported reactions).^31^ It is interesting to explore the effects of removing the supporting electrolyte on the reaction mechanism and rate (beyond the obvious benefits, such as lower cost, less waste, easy product separation, etc.). How does the absence of the supporting electrolyte affect transport, the mechanism, and the reaction zones at the interdigitated microband array electrode surface?

The absence of the supporting electrolyte introduces migration effects with effects that are dependent on the types of reactions at the anode and the cathode. The presence of an excess supporting electrolyte is important for elimination of migration (resulting in pure diffusional mass transport).^32^ However, migration can, in some cases, enhance currents^33^ and modify transport to provide beneficial effects in electrochemical processes. We can distinguish three types of chemical processes that are affected differently when the supporting electrolyte is removed from the electrochemical process and migration effects are introduced:

(1) *Charge production reactions*, which occur, for example, at anode and cathode surfaces, but could also occur in homogeneous solution (i.e., by dissociation); during charge production at the electrode surface, counter charges need to be present to maintain electroneutrality. That is, in the absence of the supporting electrolyte, local concentration gradients develop, and charge recombination is more likely to occur.
(2) *Charge neutral reactions*, which could involve ionic species reacting with neutral species or neutral–neutral reactions; these will be affected by the absence of the electrolyte due to changes in local concentrations and concentration gradients.
(3) *Charge annihilation reactions*, which are likely to occur at higher rate driven by migration in the reaction zone between anode and cathode (annihilation zone); these are accelerated by the absence of the supporting electrolyte and could be of interest in paired electrosynthesis.

In this study, the effects of an electrolyte on charge annihilation reactions are discussed. Pt–Pt interdigitated microband arrays are employed with 5 μm interelectrode spacing and 5 μm electrode width. The one-electron oxidation of 1,1′-ferrocenedimethanol is employed to characterize electrolysis currents with/without electrolyte. The two-electron two-proton reduction of activated olefins to hydrocarbon products (tetraethylethenetetracarboxylate (TET) and diethyl fumarate as models for electro-hydrogenation) is employed to explore electrosynthesis with/without an electrolyte. In contrast to conditions in the presence of an electrolyte, in the absence of the supporting electrolyte, new diffusion–migration phenomena occur, and the charge annihilation reaction between the anode and the cathode occurs at a faster rate with implications for electrosynthetic processes (although probably not affecting the reduction of activated olefins). Raman microscopy mapping is attempted for visualization of diffusion and reaction zones during electrolysis.

## 2 Experimental Section

### 2.1 Reagents

The reagents and solvents used during the experiments were purchased and used without further purification. Tetraethylethenetetracarboxylate, tetraethylethanetetracarboxylate, 1,1′-ferrocenedimethanol, LiClO_4_, diethyl fumarate, diethyl succinate, dimethyl sulfoxide (DMSO), acetone, methanol (MeOH), and ethanol (EtOH) were purchased from Merck-Sigma-Aldrich in purest or analytical reagent grade and used without further purification. Acetonitrile (MeCN) was purchased from VWR Chemicals. Deuterated solvents were obtained from Sigma-Aldrich.

### 2.2 Instrumentation

The galvanostatic or potentiostatic electrolysis method was applied with a Micro-Autolab II (Metrohm) system. Interdigitated electrodes with Pt microbands (5 μm wide microbands separated by 5 μm gaps, on a glass substrate, G-IDEPT5, Metrohm) were attached with a plastic washer by using a silicone sealant (ACC Silicones, Silicoset 15) to give a small volume microreactor for electrolysis at the 200 μL scale. Raman microscopy data were obtained with a Raman spectrometer (wavelength 785 nm excitation; with a Renishaw inVia confocal Raman microscope, 1% power). ^1^H NMR spectra were acquired on a 400 MHz Bruker Neo spectrometer equipped with an iProbe.

### 2.3 Electrolysis Procedure

Tetraethylethenetetracarboxylate (TET) and lithium perchlorate (0.1 M LiClO_4_) as electrolytes were dissolved in appropriate amounts in the solvent (methanol, MeOH, or ethanol, EtOH, or acetonitrile, MeCN). In a typical electrolysis experiment, 0.2 mL of solution was added into the cell. Cyclic voltammetry (CV) was conducted typically in a potential range of 0.5 to −2.0 V at a scan rate of 0.02 V s^–1^ with the platinum microband electrode array as both the working and counter electrodes and a silver pseudo-reference electrode (3-electrode configuration) or without the silver pseudoreference (2-electrode configuration). The galvanostatic method (constant current) was used to perform electrosyntheses. After completion of the reaction, ^1^H NMR spectroscopy (in some cases with added *d*_3_-acetonitrile, 10%) was used to determine the loss of starting material and the yield of the product.

### 2.4 Finite Element Simulation

A simplified mechanism (assuming a one-electron EC-type reduction) is considered to capture the main features with limited complexity. An anode process (BH or alcohol oxidation) and a cathode process (A reduction) are defined to produce A^–^ and H^+^ as charged species. A^–^ and H^+^ can recombine (eqs 1, 2, 3, and 4). The autoionization of the alcohol BH is considered; however, the autoionization of AH has been suppressed to allow visualization of the charge annihilation zone in terms of formation of AH from A^–^ and H^+^. Given that A^–^ is a strong base, it is likely to react with BH to give AH and B^–^ (*vide infra*). This change in mechanism causes the charge annihilation/recombination process to switch from eqs 4 to 3 in reverse.
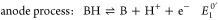
1
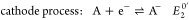
2
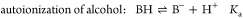
3
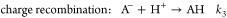
4

It is assumed that BH and A have unit activities (constant, in excess), and therefore, the transport equations are not solved for these species. Additionally, as a simplification, the transport of B is not considered. The model system consists of a rectangular geometry that represents a symmetric unit cell of the interdigitated electrode, which contains one anode at the middle with 5 μm width and 2 half cathodes at a distance of 5 μm from the anode. The height of the rectangle is 200 μm, which is 8 times higher than the diffusion layer estimated by , considering τ = 0.2 s.

#### Absence of the Supporting Electrolyte

In this case, four species are considered in the solution, of which AH is subject only to diffusion, whereas H^+^, B^–^, and A^–^ are responsible for the ionic currents (with diffusion–migration). For these species, the transport equations can be written (eqs 5, 6, 7, 8, 9, 10, 11, and 12).
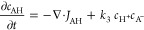
5
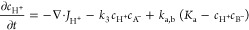
6
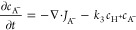
7
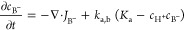
8
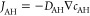
9
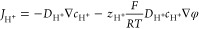
10
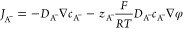
11
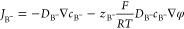
12Here, *c*_*j*_, *J*_*j*_, *D*_*j*_, and *z*_*j*_ are the instantaneous local concentration, mass flux, diffusion coefficient, and ionic charge of species *j*, respectively, knowing that *j* represents species AH, H^+^, A^–^, and B^–^. *F* and *R* are the Faraday constant and the ideal gas constant. *T* is the absolute temperature. φ is the instantaneous local electrolyte potential, *k*_3_ is the rate constant for the formation of AH, *k*_a,b_ is the rate constant for the alcohol association reaction, and *K*_a_ is the alcohol dissociation equilibrium constant. Additionally, since the migration transport is mediated by the electric field, which in its turn depends on the ionic concentrations, it is necessary to write Poisson eq 13 with ε_r_ as the relative permittivity of the alcohol and with the vacuum permittivity, ε_0_.
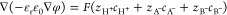
13

The initial concentrations for the species H^+^ and B^–^ were obtained from the equilibrium of eq 3, knowing that the *K*_a_ is 3.16  × 10^–16^;^34^ then, they are 17.8 nmol L^–1^, whereas, for A^–^ an initial concentration of 1 pmol L^–1^ was admitted, and AH was considered to be initially absent. The electrolyte potential at the beginning of the simulation was set to zero. Simulations were performed assuming a constant applied voltage (chronoamperometry), which was selected to reach a predefined quasi-steady-state current. At the anode, fluxes of production of H^+^ at the anode and A^–^ at the cathode are given by the faradic current related to the eqs 1 and 2, respectively. For all of the other species, the boundary fluxes are null because they do not participate in any redox process, as shown in Table 1.

**Table 1:** Boundary Conditions for eqs 5–8

boundary	condition
anode	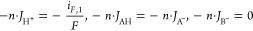
cathode	
gap and mass transport domain border	

Here, *i*_*F*,1_ and *i*_*F*,2_ are the local faradic current densities at the anode and cathode surfaces, respectively, defined by
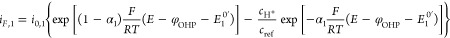
14
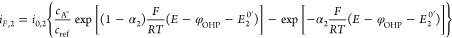
15

The parameters *i*_0,*j*_, α_*j*_, and *E*_*j*_^0*′*^ are the exchange current density, the transfer coefficient, and the formal potential, respectively; for the reaction *j*, *E* is the applied potential, φ_OHP_ is the electrolyte potential at the outer Helmholtz plane, and *c*_ref_ is a reference concentration, which was chosen to be 1 mol L^–1^. The boundary condition^35^ at the electrode surface for the eq 13 is given by
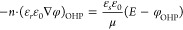
16where *ε*_S_ and μ are the relative permittivity and the thickness of the Stern layer. The value of μ depends on the electrolyte composition, and it was estimated by the following expression if the supporting electrolyte properties are disregarded.
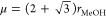
17

At the insulate surfaces, the boundary condition^36^ for the eq 13 is
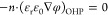
18

The parameter *r*_MeOH_ = 0.18 nm is the radius of the methanol molecule.^37^

### Presence of the Supporting Electrolyte

Two further transport equations must be added to the model to take into account the transport of the electrolyte ions of C^+^ and D^–^.
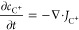
19
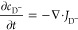
20
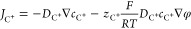
21
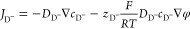
22

The parameters in the equations above have the same meanings as those stated before. The parameters *z*_H^+^_, *z*_A^–^_, and *z*_B^–^_ were set to zero (only for the case of the presence of the electrolyte) because the migration effects were not taken into account. The source term in eq 13 needed to be rewritten as eq 23.
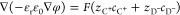
23

The initial conditions for the electrolytes *c*_C^+^_ and *c*_D^–^_ were set as 0.1 mol L^–1^, and the fluxes of C^+^ and D^–^ are zero at any boundary. The amount of these ions remains constant during the entire simulation. The parameters employed in the simulations are summarized in Table 2.

**Table 2:** Summary of Parameters Used in the Simulation

symbol	value	description
*i*_0,1_	1000 A m^–2^^a^	anodic exchange current density
*i*_0,2_	1000 A m^–2^^a^	cathodic exchange current density
α_1_	0.5^a^	anodic transfer coefficient
α_2_	0.5^a^	cathodic transfer coefficient
*E*_1_^0′^	0 V^a^	anodic formal potential
*E*_2_^0′^	0 V^a^	cathodic formal potential
*K*_a_	3.16 × 10^–16^ (or 10^–15.5^)	alcohol dissociation equilibrium constant^34^
*k*_a,b_	4 × 10^7^ m^3^ s^–1^ mol^–1^^a^	rate constant for the alcohol association reaction
*k*_3_	8 × 10^7^ m^3^ s^–1^ mol^–1^^a^	rate constant for the formation of AH
*D*_H^+^_	2.34 × 10^–9^ m^2^ s^–1^^38^	diffusion coefficient of hydronium
*D*_A^–^_	1.00 × 10^–9^ m^2^ s^–1^^a^	diffusion coefficient of A^–^
*D*_B^–^_	1.00 × 10^–9^ m^2^ s^–1^^a^	diffusion coefficient of B^–^
*D*_AH_	1.00 × 10^–9^ m^2^ s^–1^	diffusion coefficient of AH
*D*_C^+^_	1.10 × 10^–9^ m^2^ s^–1^^39^	diffusion coefficient of C^+^
*D*_D^–^_	1.38 × 10^–9^ m^2^ s^–1^^37^	diffusion coefficient of D^–^
ε_r_	32.7^40^	relative permittivity in the bulk
ε_s_	11^a^	relative permittivity in the Stern layer
μ	6.72 × 10^–10^ m	thickness of the Stern layer
*C*_s_		capacitance of the Stern layer

a Parameters with arbitrarily chosen values (not accessible experimentally).

In the analysis of the charge annihilation process, a second-order process is assumed, governed by the concentration of cations and anions (here, H^+^ and A^–^) in a particular location. The rate of the second-order charge annihilation process is estimated based on the product of concentrations for H^+^ and A^–^ integrated over space (parameter Ψ in eq 24), assuming (at least in the first approximation) that the corresponding rate constant *k*_3_ (diffusion controlled) is not significantly affected by the electrolyte and activity effects.
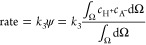
24

These equations are solved by employing the finite element method (FEM, COMSOL Multiphysics 6.0). A mesh was built according to a predefined method with a maximum elemental size of 2.01 μm. At the boundary that contains the electrodes, the maximum element size was 10 nm. This was necessary to ensure accurate calculation of the gradients in every region. The total mesh in the model was constituted of about 72,790 elements. The computational time spent in the simulation of each of the conditions was about 2.2 h (on a personal computer with a processor Intel Core I7-7800X with 6 cores working at 3.50 GHz and 128 GB of RAM memory).

## 3 Results and Discussion

### 3.1 Electrosynthesis at Interdigitated Electrodes I.: Voltammetry

**Figure 1:**
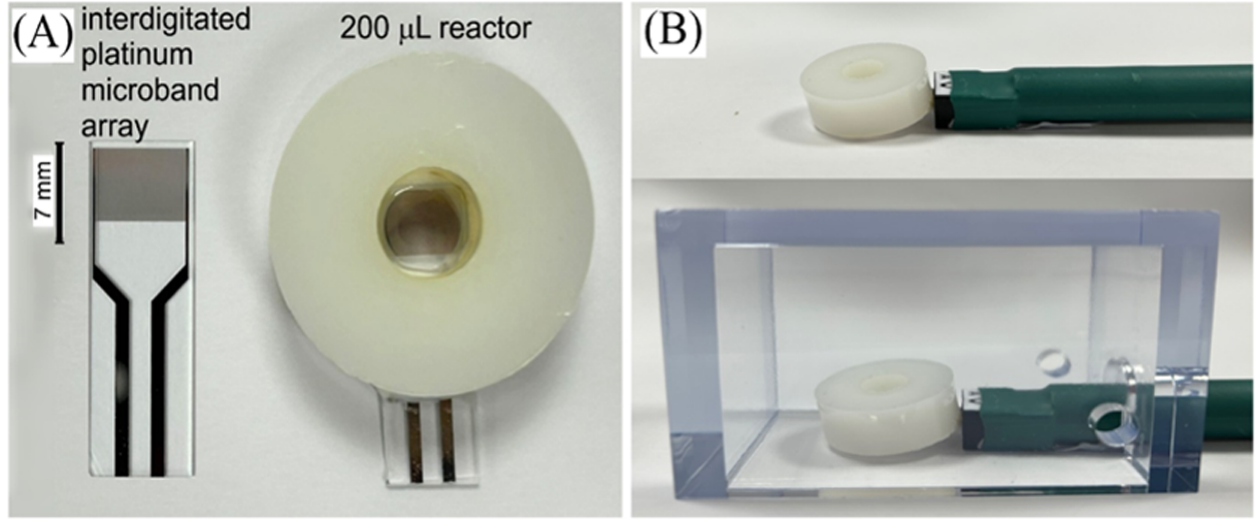
Photographs of (A) the interdigitated Pt microband array electrode and the microelectrosynthesis cell and (B) the cable attachment and 3D-printed housing.

In this report, electrochemical processes at interdigitated electrodes are investigated, and 1,1′-ferrocenedimethanol and tetraethylethenetetracarboxylate (TET) are chosen as model redox systems. A commercial platinum microband interdigitated array electrode was employed with Pt bands of 5 μm width and 5 μm separation. The resulting active geometric field of electrodes has an area of 5 mm × 6 mm (total width × length of bands) = 0.3 cm^2^. There are 250 microbands in each of the two electrodes. A washer (5 mm high) was used, attached to an electrode to construct a microreaction cell (Figure 1A). This electrolysis cell can be used with typically a 0.2 mL volume of liquid in a three-dimensional (3D)-printed housing (Figure 1B) to control the environment.

In order to explore reactivity in the microelectrolysis cell, the chemically reversible one-electron oxidation of 1,1′-ferrocenedimethanol was used. Equation 25 shows a reversible electrochemical process (E-mechanism).
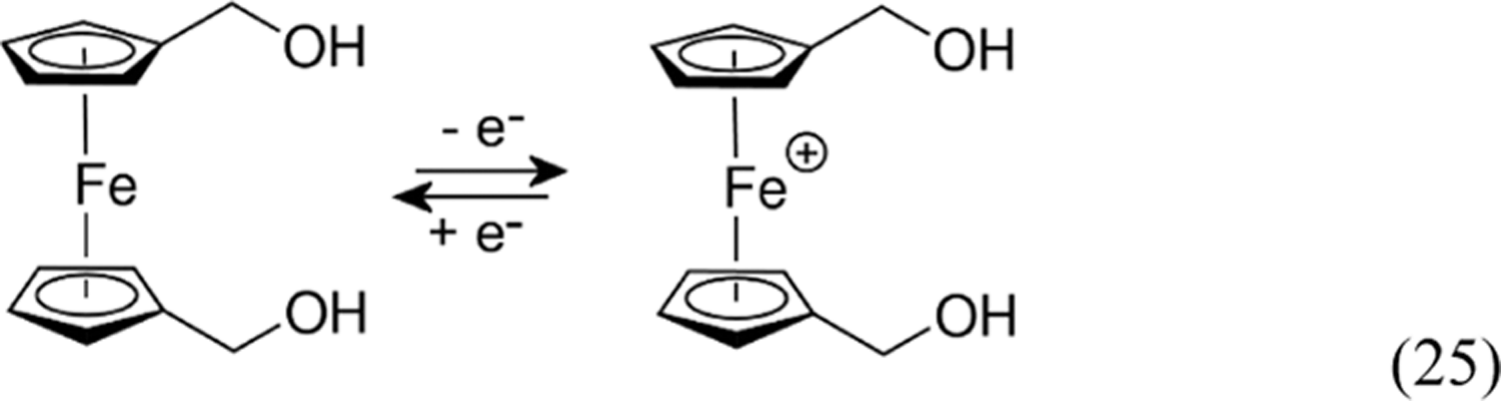
25

**Figure 2:**
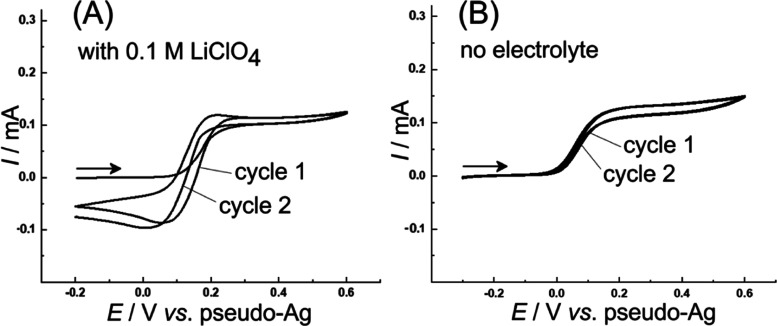
(A) Cyclic voltammograms (scan rate 0.01 V s^–1^; first and second potential cycle) for the oxidation of 1 mM 1,1′-ferrocenedimethanol in methanol containing 0.1 M LiClO_4_ electrolyte. (B) As before, but without intentionally added electrolyte (note that the working electrode and the counter electrode are in close vicinity).

Figure 2 shows cyclic voltammetry data recorded with a silver wire pseudo-reference electrode and by using the two Pt microband arrays as the working and the counter electrodes. When the applied potential is scanned from the range of zero current (Fe(II) is stable), an oxidation response is observed at 0.15 V vs *pseudo*-Ag consistent with the oxidation of the 1,1′-ferrocenedimethanol into the Fe(III) state.^41^ The current due to 1,1′-ferrocenedimethanol redox cycling plateaus at approximately 0.1 mA.

The theory for redox cycling at interdigitated microband array electrodes has been reviewed by Aoki,^42^ and the equation for the special case of electrode width = interelectrode gap is given here in good approximation (the constant multiplier simplified to 1.0) in eq 26.
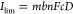
26

**Figure 3:**
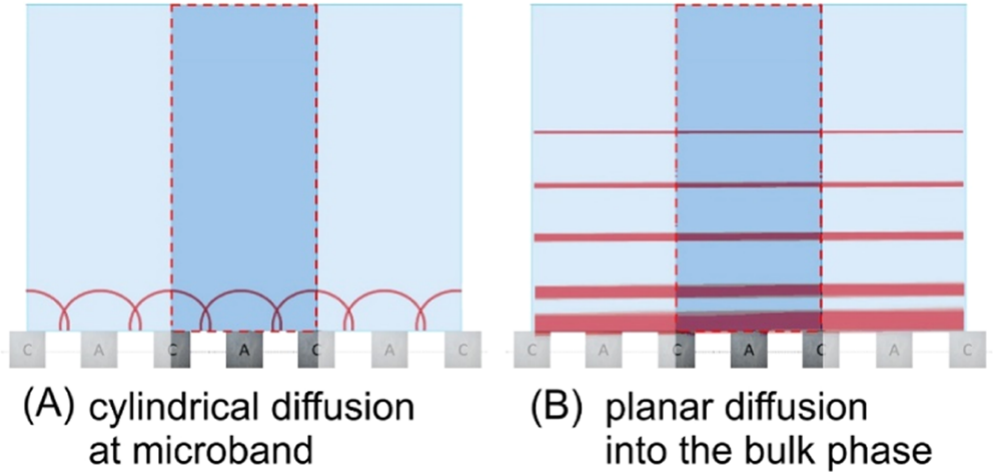
Illustration of (A) local cylindrical feedback diffusion between microband electrodes and (B) approximately planar diffusion for reactants that are irreversibly converted to products (no redox cycling).

In this expression, *m* = 250 is the number of individual band electrodes (for both the anode and the cathode), *b* = 6 × 10^–3^ m is the approximate length of bands, *n* = 1 is the number of electrons transferred per molecule diffusing to the electrode, *F* is the Faraday constant, *c* is the bulk concentration, and *D* is the diffusion coefficient for the redox active species. For 1,1′-ferrocenedimethanol, the diffusion coefficient in water was reported as 0.6 × 10^–9^ m^2^ s^–1^.^43^ Employing the relationship between diffusion coefficient and dynamic viscosity, *D* ∼ 1/η (using the Stokes–Einstein equation),^44^ with η(methanol) = 0.54 mPa s and η(water) = 0.89 mPa s, an estimate for the diffusion coefficient in methanol is obtained as *D* = 1.0 × 10^–9^ m^2^ s^–1^. The calculated quasi-steady-state current for 1 mM 1,1′-ferrocenedimethanol is *I*_lim_ = 0.14 mA. This is slightly higher compared to the limiting current observed in Figure 2A (in the presence of 0.1 M LiClO_4_), possibly due to not all oxidized species being reduced back to the Fe(II) state (i.e., some oxidized 1,1′-ferrocenedimethanol diffuses into the bulk). In fact, when investigating the second potential cycle in this experiment, both Fe(II) and Fe(III) are present, and limiting currents are observed for both oxidation and reduction indicative of Fe(III) bulk product formation. The detection of the Fe(III) product in the second potential cycle suggests predominantly cylindrical diffusion with a contribution of planar diffusion into the bulk solution (Figure 3).

In the absence of an intentionally added electrolyte (Figure 2B), the oxidation of 1,1′-ferrocenedimethanol occurs at 0.1 V vs pseudo-Ag with a limiting current of 0.13 mA, apparently consistent with eq 26. In the second potential cycle, the reduction of Fe(III) in solution (produced in the previous potential cycle) is not observed. It seems likely that monocationic Fe(III) species produced under these conditions escape to a lesser extent into the bulk solution (less planar diffusion due to enhanced migration). These cations are forced by migration to react back to Fe(II) at the cathode (enhanced cylindrical diffusion–migration). The transport pathway and the reaction zone appear to change due to the absence of a supporting electrolyte, although details, in particular, for the cathode process, are currently not well known. Next, the two-electron reduction of tetraethylethentetracarboxylate (TET, eq 27) is considered as a chemically irreversible EC-type redox process.
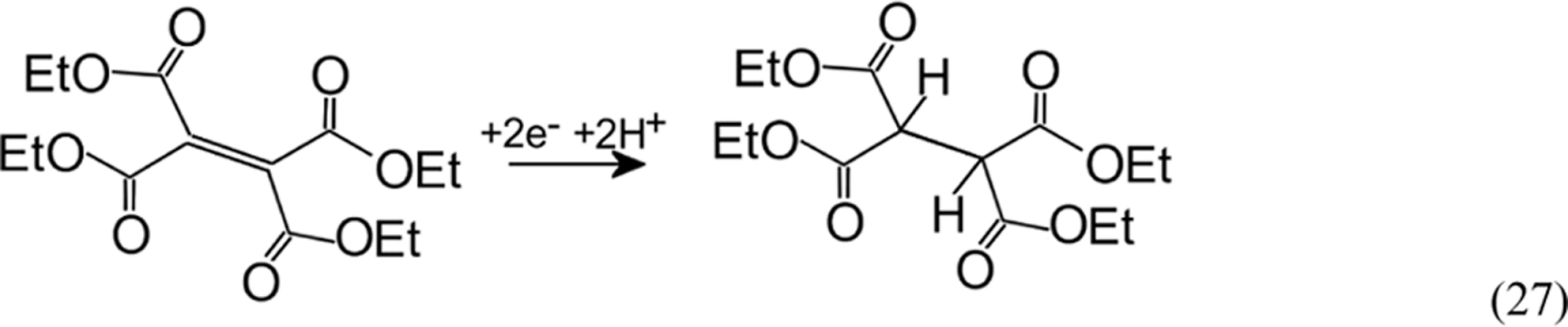
27

**Figure 4:**
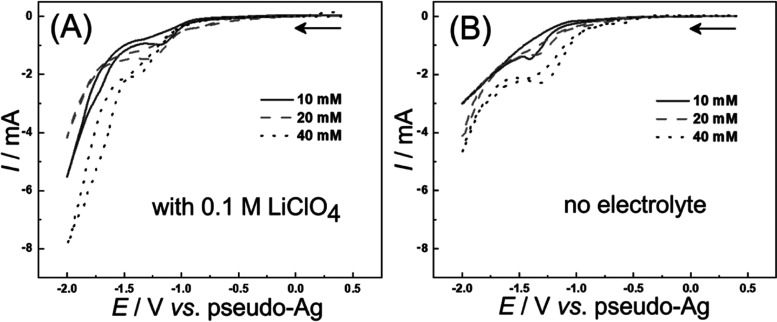
(A) Cyclic voltammograms (scan rate 0.02 V s^–1^) for the reduction of 10, 20, or 40 mM TET in methanol containing 0.1 M LiClO_4_ electrolyte. (B) As before, but without an intentionally added electrolyte (note that here, the working electrode and the counter electrode are the microbands in close vicinity).

Figure 4 shows cyclic voltammetry data for the reduction of tetraethylethenetetracarboxylate (TET) in methanol with a 0.1 M LiClO_4_ electrolyte. A peak feature at −1.3 V vs pseudo-Ag is followed by a further current increase at more negative applied potentials. The peak current increase is linked to the increase in solution concentration of TET, although not in a linear manner.

The peak current is again in the mA range. A two-electron conversion associated with protonation to give the electrochemically inactive tetraethylethanetetracarboxylate (H_2_TET) is likely. When employing the Randles–Sevcik equation^45^ (eq 28) to roughly estimate the peak current for this process (assuming approximately planar diffusion from the bulk and assuming consumption of olefin at the array, ignoring the fact that the irreversibility of the chemical process can slightly alter the peak current; Figure 3B), a reasonable match in terms of currents is obtained.
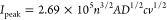
28

In this equation, the approximate peak current *I*_peak_ = 1.0 mA is linked to the number of electrons transferred per molecule diffusing to the surface *n* = 2, the total geometric area *A* = 30 × 10^–6^ m^2^, an estimated *D* = 1.0 × 10^–9^ m^2^ s^–1^, *c* = 10 mol m^–3^, and the potential scan rate *v* = 0.02 V s^–1^. Peak currents do increase with scan rate in support of this interpretation of the current (for sufficiently long time scales approximately planar diffusion), with further support from bulk electrolysis (*vide infra*). In the absence of the supporting electrolyte (Figure 4B), very similar current responses are observed, with the background current (probably linked to hydrogen evolution) being pushed out to more negative potentials. Although the current responses are similar, it is likely that the absence of the supporting electrolyte modifies the transport pathways and the reaction zones during olefin electro-hydrogenation changes (*vide infra*).

**Figure 5:**
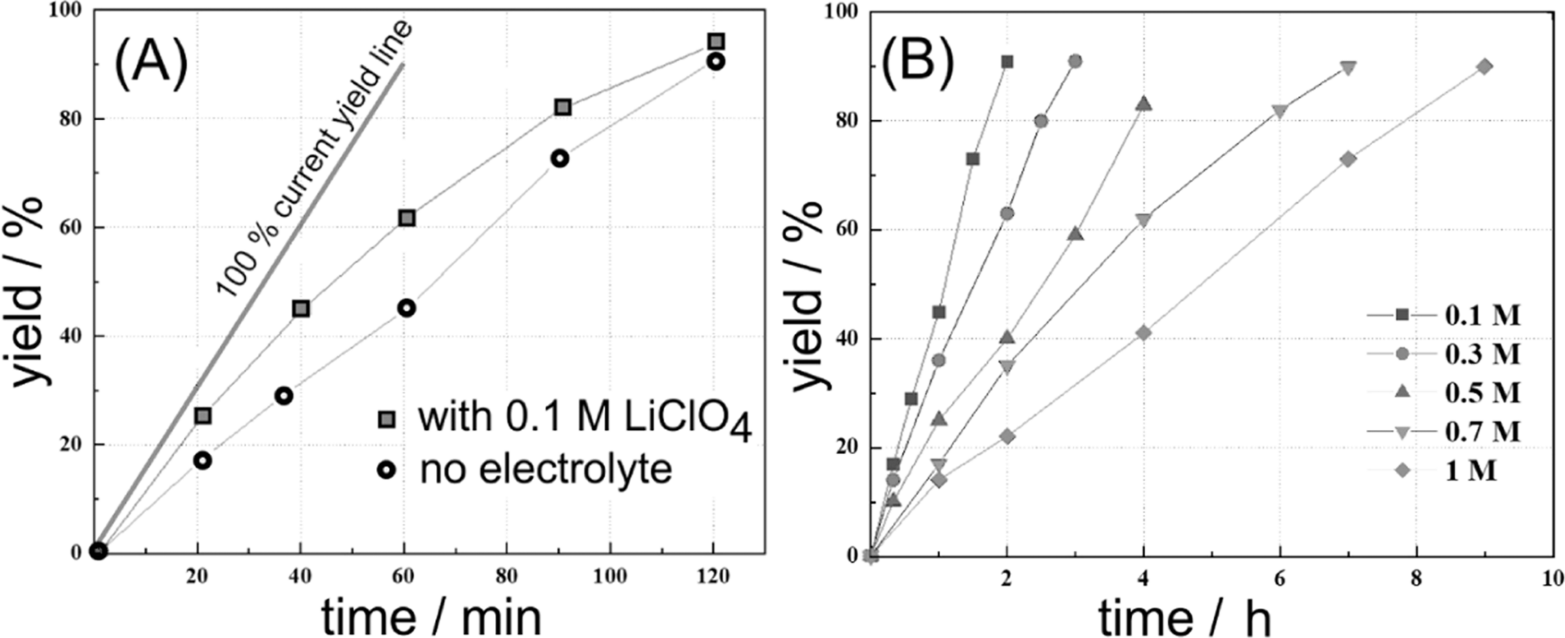
(A) Plot of tetraethylethanetetracarboxylate (H_2_TET) yields versus synthesis time under the same experimental condition (0.1 M TET solution, 200 μL, in MeOH, 1 mA current, galvanostatic) with/without lithium perchlorate (LiClO_4_). (B) Plot of yield versus synthesis time employing different TET concentrations (0.1, 0.3, 0.5, 0.7, and 1.0 M; in MeOH; galvanostatic 1 mA current, without the electrolyte). Yields are based on ^1^H NMR data (experiments were stopped; the 200 μL sample was mixed with 100 μL CD_3_CN and 300 μL MeOH; the yield was calculated from the ^1^H NMR signal for the −CH_2_– regions for the ethyl ester).

In bulk electrolysis experiments, the cell is operated in two-electrode mode, and both starting material consumption and product yields are investigated as a function of time. Figure 5A shows the formation of the product tetraethylethanetetracarboxylate (H_2_TET) as a function of electrolysis time (for a 1 mA electrolysis current; galvanostatic). For both the presence and absence of 0.1 M LiClO_4_ electrolyte, good yields are observed. The maximum yield (based on ^1^H NMR data; theoretical current yield calculated by assuming the two-electron conversion in eq 3) is indicated by a line. In the first 30 min of the process, the reaction is close to the theory line indicative of very effective electrolysis. In fact, the electrolysis in the absence of the supporting electrolyte seems just as effective as the process in the presence of 0.1 M LiClO_4_. Losses due to electron shuttling between the cathode and the anode seem negligible (indicative of an EC-type process for reduction close to the cathode). Note that higher concentrations of substrate up to 1 M are readily employed to decrease the need for a solvent.

In order to explain the high efficiency even in the absence of a supporting electrolyte and the decay in efficiency for longer electrolysis times, the transport processes in the microreactor cell have to be considered. In the 8 mm diameter cell (Figure 1), a volume of 200 μL is consistent with a fill height of approximately 2 mm. The steady-state nature of the limiting current response in Figure 4 suggests the presence of a diffusion layer (associated with some natural convection). The Nernst diffusion layer model (eq 29) can be employed to express the diffusion layer.
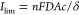
29

For the conditions in Figure 4 and using an approximate value for *D* = 1.0 × 10^–9^ m^2^ s^–1^ for TET, we obtain the diffusion layer thickness of approximately δ = 100 μm. Beyond this planar diffusion layer, we can consider the solution to behave as a bulk reservoir. As bulk electrolysis progresses, the concentration *c* in the bulk will decrease, and at some point, the applied current will be higher than the flux of starting material to the electrode surface (given by eq 29). At this point, the current efficiency decays (side reactions such as solvent breakdown occur), as seen in Figure 5A. When increasing the concentration of TET, the current efficiency remains high, but the electrolysis time at constant current increases (Figure 5B). The counter electrode reaction can be attributed to sacrificial methanol oxidation and formation of formaldehyde and protons (balanced by the cathodic process). Data in Table 3 summarize electrosynthesis experiments in different solution environments. Protic solvents such as methanol and ethanol produce the reduced olefin in a good yield. Acetone and acetonitrile pure are not effective; however, with addition of a proton source, they can be employed.

**Table 3:** Summary of Data for Galvanostatic TET Reduction to H_2_TET in the Absence of a Supporting Electrolyte and with Different Proton-Donors Added

entry	H donor/solvent	concentration	current	time	yield^a^
1	MeOH	0.1 M	1 mA	1 h	45%
2	EtOH	0.1 M	1 mA	1 h	59%
3	MeCN	0.1 M	1 mA	1 h	0%
4	MeCN with acetic acid 0.05 M	0.1 M	1 mA	1 h	36%
5	DMSO (2% H_2_O)	0.1 M	1 mA	1 h	25%
6	acetone (1% H_2_O)	0.3 M	1 mA	2 h	3%

a The yield was calculated according to ^1^H NMR data.

Data in Table 4 show that less activated olefins such as fumarate (eq 30) can be reduced under similar conditions but only when present in a high concentration (1 M) and when employing higher current densities. The electrochemical reduction of the less activated diethyl fumerate occurs at a more negative electrode potential and, therefore, closer to the solvent decomposition (hydrogen evolution) background. This may lower the yield.
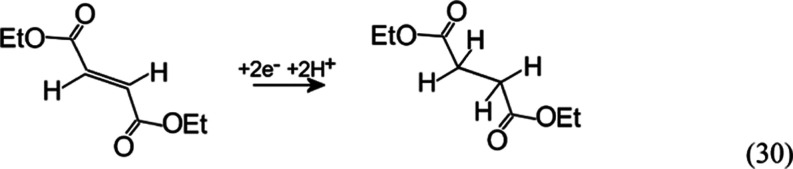
30

**Table 4:** Summary of Data for Galvanostatic Diethyl Fumerate Reduction to Diethyl Succinate in Methanol in the Absence of a Supporting Electrolyte

entry	concentration	current	time	loss of starting material^a^	yield of product^a^
1	1.0 M	10 mA	2 h	90%	70%
2	0.3 M	1 mA	2 h	28%	12%^a^
3	0.1 M	1 mA	1 h	23%	5%^a^
4	0.1 M	1 mA	2 h	36%	8%^a^
5	0.1 M	1 mA	3 h	48%	12%^a^

a The conversion and yield were calculated according to ^1^H NMR data.

### 3.2 Electrosynthesis at Interdigitated Electrodes II.: In Situ Raman

**Figure 6:**
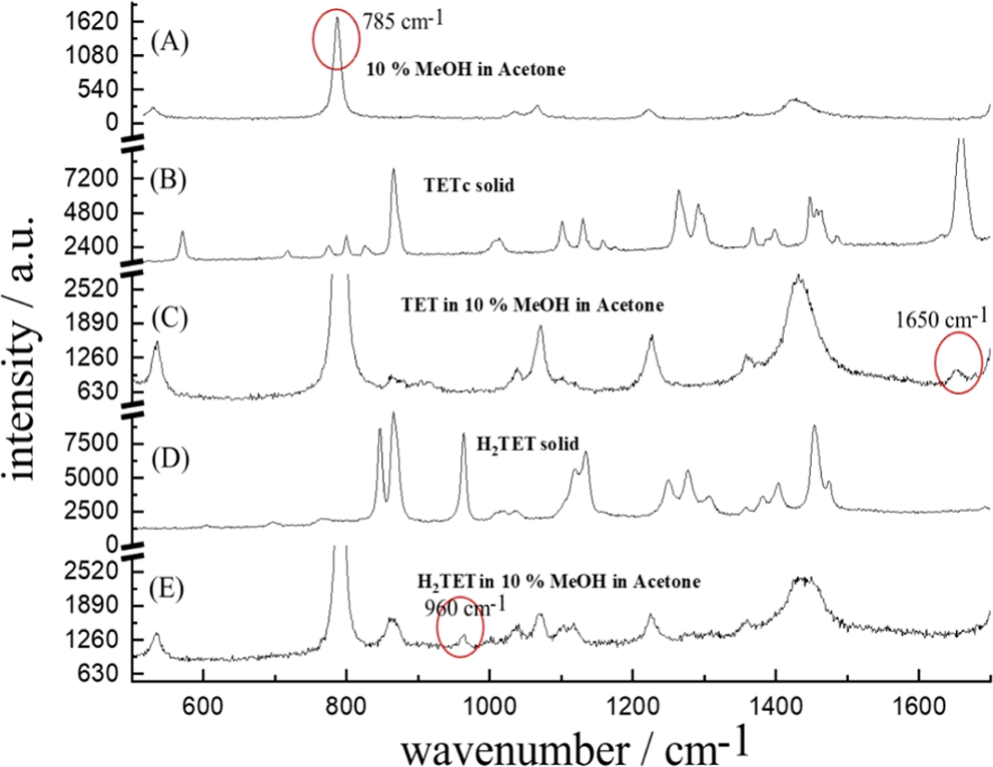
Raman data for solutions in 10% MeOH/acetone. (A) Solvent background. (B) TET starting material as solid. (C) TET starting material in solution with a diagnostic band at 1650 cm^–1^. (D) H_2_TET product as a solid. (E) H_2_TET product in solution with a diagnostic band at 960 cm^–1^.

In order to investigate reaction zones during electrolysis, an in situ Raman spectroscopy/microscopy experiment was devised.^46^ Due to the low sensitivity of Raman, the solvent system was optimized to 10% methanol in acetone to allow approximately 1.0 M TET and 1.0 M H_2_TET solutions for better Raman detection. Figure 6 shows data for the solvent, the solids, and the solutions. Two peaks at 1650 and 960 cm^–1^ are specific for the olefin starting material and for the reduction product, respectively.

**Figure 7:**
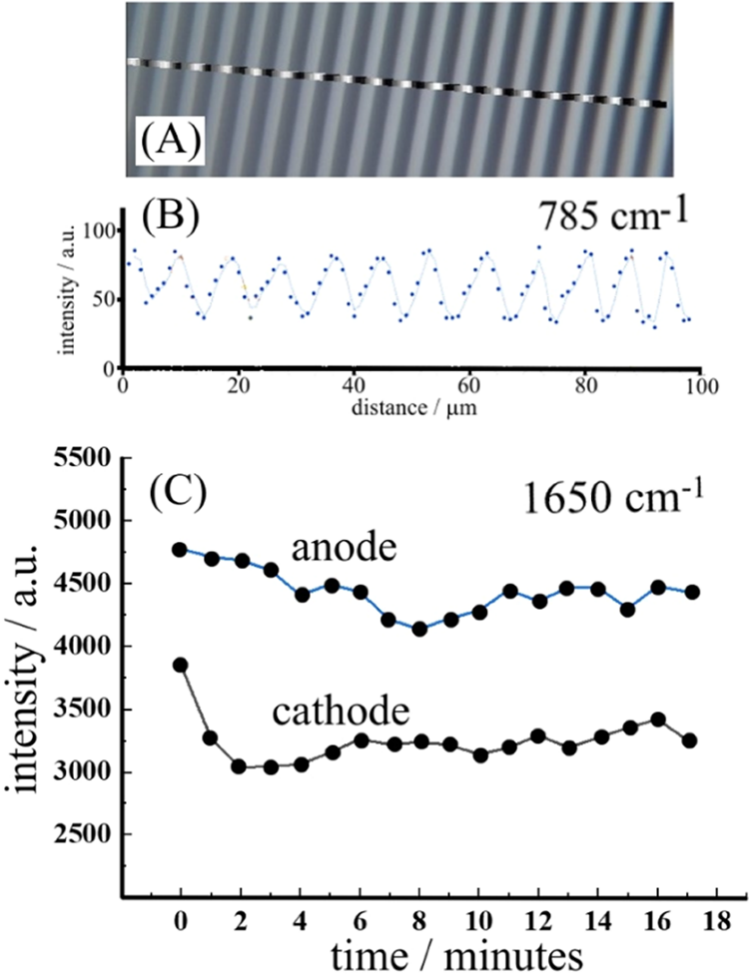
Raman microscopy data showing (A) a scan over the interdigitated microband array and (B) the corresponding change in the solvent band intensity (mainly due to reflection from the platinum surface enhancing the Raman signals). (C) Time-dependent data (stepping the applied current to −1 mA at time = 0 min) obtained on the anode and on the cathode for the starting material TET band.

A scan over the electrode array surface shows a significant intensity variation (Figure 7A), enhanced by reflection from the platinum band electrodes. When placing the laser beam onto the cathode (785 nm wavelength; approximately 2 μm diameter spot size; probing approximately 2 μm from the surface; Figure 7C cathodic) and monitoring the peak at 1650 cm^–1^, a distinct loss of intensity with electrolysis time is observed in the first 2 min of electrolysis, consistent with planar diffusion supplying the TET starting material to the electrode surface. The laser probes the region above the cathode and is, therefore, most sensitive to TET depletion. Figure 7C also shows data for the liquid phase in contact with the anode, where only a minor slow loss of intensity of the TET band at 1650 cm^–1^ implies some bulk depletion. The product band at 960 cm^–1^ can be observed but is too weak to give reliable time transient data. In summary, Raman microscopy data confirm the depletion of TET (over the cathode) and formation of H_2_TET. However, the intensity of signals is currently too low to map the reaction zones in more detail.

### 3.3 Electrosynthesis at Interdigitated Electrodes III.: Mechanism

**Figure 8:**
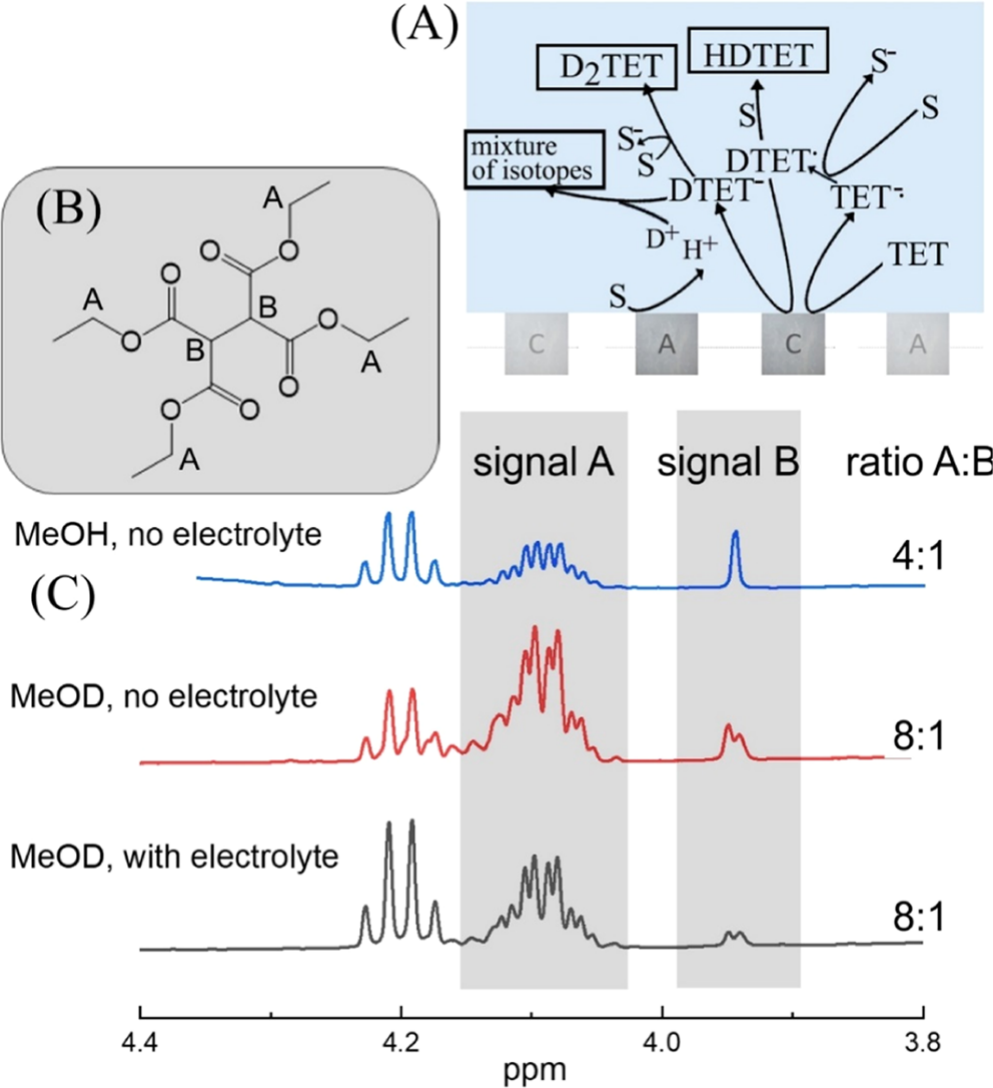
(A) Illustration of kinetic pathways. (B) Molecular structure. (C) ^1^H NMR data for electrolysis in MeOH (signal A is attributed to the −O–CH_2_– moiety of ethyl groups; note the quartet on the left due to the same protons in the starting material; signal B is attributed to C–H formed during reduction) and in MeOD with/without added 0.1 M LiClO_4_.

To investigate the mechanism of the TET reduction reaction (and the relevance of the charge annihilation process), CH_3_OD solvent is employed instead of CH_3_OH. The isotope effect (see eq 31) could lead to D_2_TET (bis-deuteration in the case of formation of anions at the cathode, followed by protonation with CH_3_OD), or it could lead to HDTET (monodeuteration in the case of initial formation of an olefin anion D^+^ transfer from CH_3_OD, followed by H-radical abstraction from the solvent; in cathode reaction zone; eq 31). Due to anodic formation of a 50:50 mixture of D^+^ and H^+^ due to solvent oxidation to formaldehyde, there is also a possibility of H^+^ and D^+^ transferring toward the cathode to produce a mixture of H_2_TET, HDTET, and D_2_TET (formed in the charge annihilation zone). Figure 8A summarizes the alternative pathways for this reaction and the diagnostic reaction products. Data analysis is performed with ^1^H NMR.
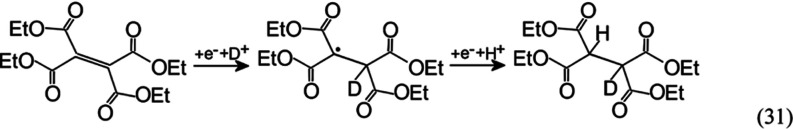
31

Figure 8B,C shows ^1^H NMR data for the 4.10 ppm region where the −O–CH_2_– protons of the ethyl groups are observed (signal A; note the quartet on the left for the same protons in the starting material). In the 3.95 ppm region, the C–H (signal B) of the reduced olefin is detected. Signal A is unexpectedly complex (multiple quartets) due to rotamers being resolved at 400 MHz. The electrochemical reduction in MeOH leads to the expected ratio A/B of 4:1. When performed in MeOD, the same process leads to a ratio A/B of 8:1. Therefore, the first electron transfer is followed by an initial fast deuteration step. The resulting radical is then likely to be able to extract an H atom from the solvent (product HDTET). Alternatively (less likely), the result could be interpretated in terms of H^+^/D^+^ being formed at the anode in a 50:50 ratio to cause the observed isotope ratio of the product formed at the cathode (producing a mixture of H_2_TET, HDTET, D_2_TET). In this case, the presence/absence of the electrolyte should impact the degree of deuteration, but it does not. Data in Figure 8 show that the presence or absence of the supporting electrolyte does not affect the outcome of the TET reduction process. In both cases, the product is HDTET. Data in Table 5 summarize the reaction conditions. Next, in order to better understand the reaction conditions and reaction zones, a finite element simulation is performed for a simplified mechanism.

**Table 5:** Summary of Data for Galvanostatic TET Reduction to *d*_2_-Tetraethylethane-tetracarboxylate (D_2_TET) or to *d*-Tetraethylethane-tetracarboxylate (HDTET) in CH_3_OD (99% Deuterated) in the Absence of a Supporting Electrolyte

H donor/solvent	TET concentration	current	time	electrolyte	product yield^a^
MeOH	0.1 M	1 mA	1.3 h	none	55%
MeOD	0.5 M	1 mA	4 h	none	73% (with 50% deuteration)
MeOD	0.5 M	1 mA	4 h	0.1 M LiClO_4_	60% (with 50% deuteration)

a The yield was calculated according to ^1^H NMR data.

### 3.4 Electrosynthesis at Interdigitated Electrodes IV.: Finite Element Simulation of Reaction Zones

In order to better (quantitatively) rationalize and understand reaction zones and charge annihilation processes in the absence of the supporting electrolyte, finite element simulation has been carried out. Only a simplified mechanism is considered (with A = olefin and BH = methanol) based on a cathode reaction (A + e^–^ ⇄ A^–^), an anode reaction (BH ⇄ B + H^+^ + e^–^), a homogeneous charge annihilation step (A^–^ + H^+^ ⇄ AH), and a homogeneous equilibrium process (BH ⇄ B^–^ + H^+^). Although not representing all aspects of the mechanism, this subset of reactions allows effects from the supporting electrolyte to be visualized. The proton-transfer reaction with the solvent (A^–^ + BH ⇄ AH + B^–^) has been ignored for clarity. The impact of this will be discussed in the context of charge annihilation reactions. Crucial questions are (i) how does the absence of an electrolyte modify the transport path of products formed at the anode and cathode, and (ii) what impact does the absence of an electrolyte have on the homogeneous reactions and reaction zones coupled to the oxidation and reduction?

**Figure 9:**
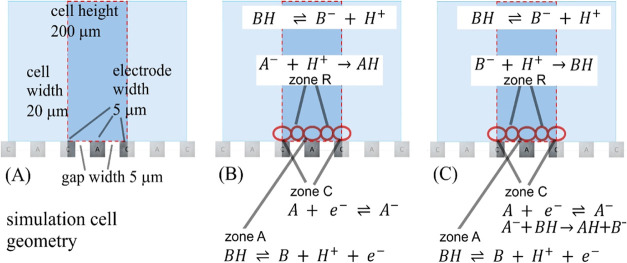
Simulation model: (A) geometry of the 2D simulation cell, (B) oversimplified as used in the simulation, and (C) more realistic (not implemented in the simulation) based on interdigitated anodes and cathodes with reaction zones (see the text).

Figure 9 shows the schematic for the finite element model (two-dimensional (2D) cell with 20 μm × 200 μm size; illustrated by the dark blue region with the anode and the cathode indicated) with reaction zones. Zone A at the anode is linked to the oxidation of BH (the methanol solvent), and zone C at the cathode is linked to the reduction of A. The products from these two processes, A^–^ and H^+^, are assumed to undergo diffusion–migration to finally interact and react in charge annihilation (zone R). Transport into this zone is likely to be most affected by the presence/absence of an electrolyte.

**Figure 10:**
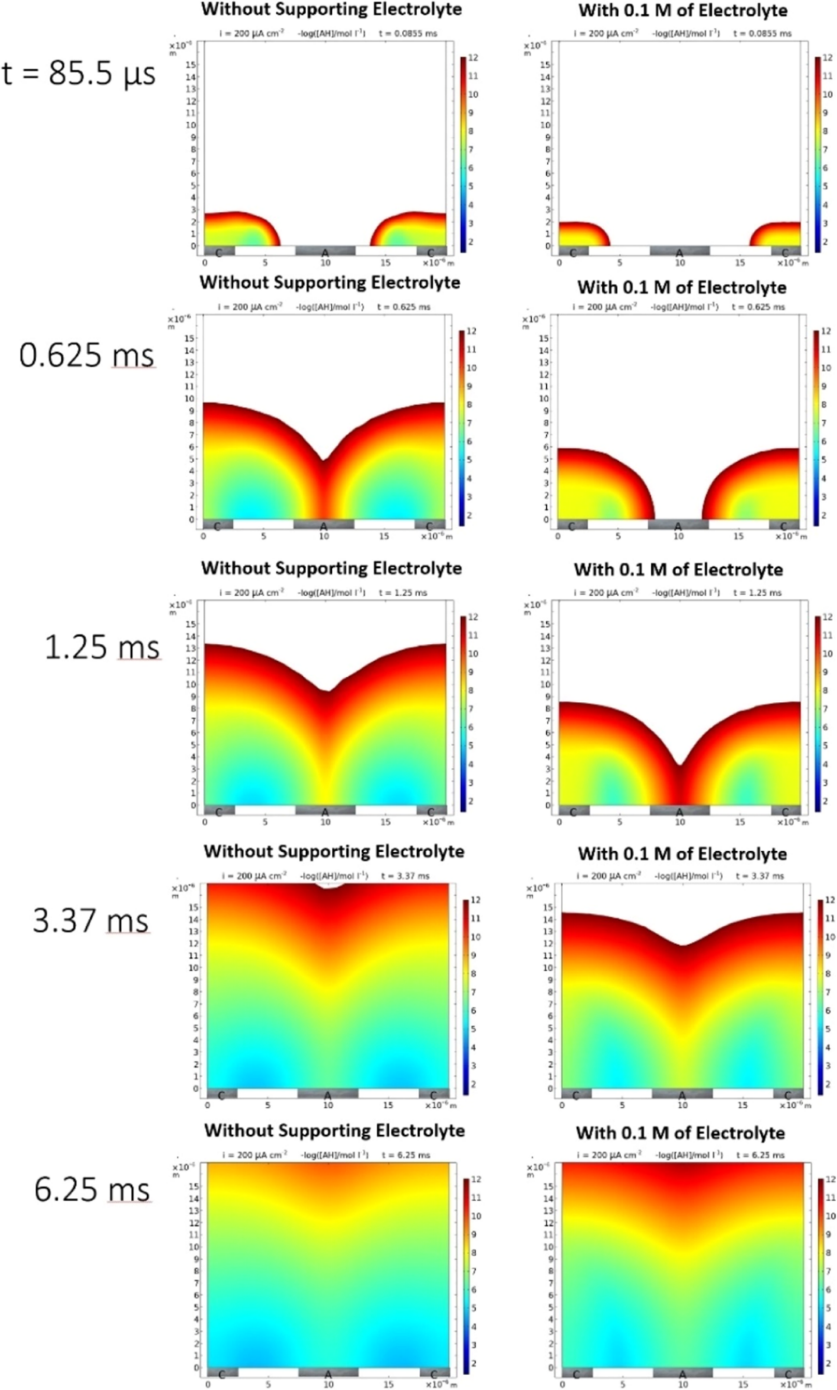
Plots of concentration of AH (the product from charge annihilation, see Figure 9B) in the region around the anode and the cathode and as a function of time. The left *y*-axis gives the distance (in μm) from the electrode surface, and the right *y*-axis shows the color coding in terms of the negative decadic exponent for concentration (i.e., dark red = 10^–12^ mol dm^–3^ and dark blue = 10^–2^ mol dm^–3^). Plots for other species are provided in the Supporting Information.

In the finite element simulation, a constant voltage is imposed to lead to quasi-steady-state currents due to processes at the anode and the cathode. Reaction zones A and C represent regions where anodic and cathodic reactions occur. Reaction zone R represents the region of interest for the interaction of anode and cathode products and for charge annihilation. Figure 10 shows plots of the AH concentration as a function of time. Initially, AH is formed due to reduction of A at the cathode (zone C). The solvent methanol is sufficient to provide protons for the formation of AH. However, the rate of BH autoionization is limited. Following an initial phase of AH production in zone C, A^–^ can diffuse toward the anode, where protons are generated for the annihilation process. At a time of 0.625 ms, the diffusion–migration transport zones of A^–^ and H^+^ overlap, and regions of high AH production are observed between the anode and the cathode (annihilation reaction in zone R). Perhaps interestingly, this process is much faster (more effective) in the absence of the supporting electrolyte. At a time of 1.25 ms, an order of magnitude increase in AH production is observed in the absence of the supporting electrolyte when compared to data with an electrolyte. This pattern continues and is related to the migration term accelerating the transport of oppositely charged ions toward each other for the charge annihilation process.

**Figure 11:**
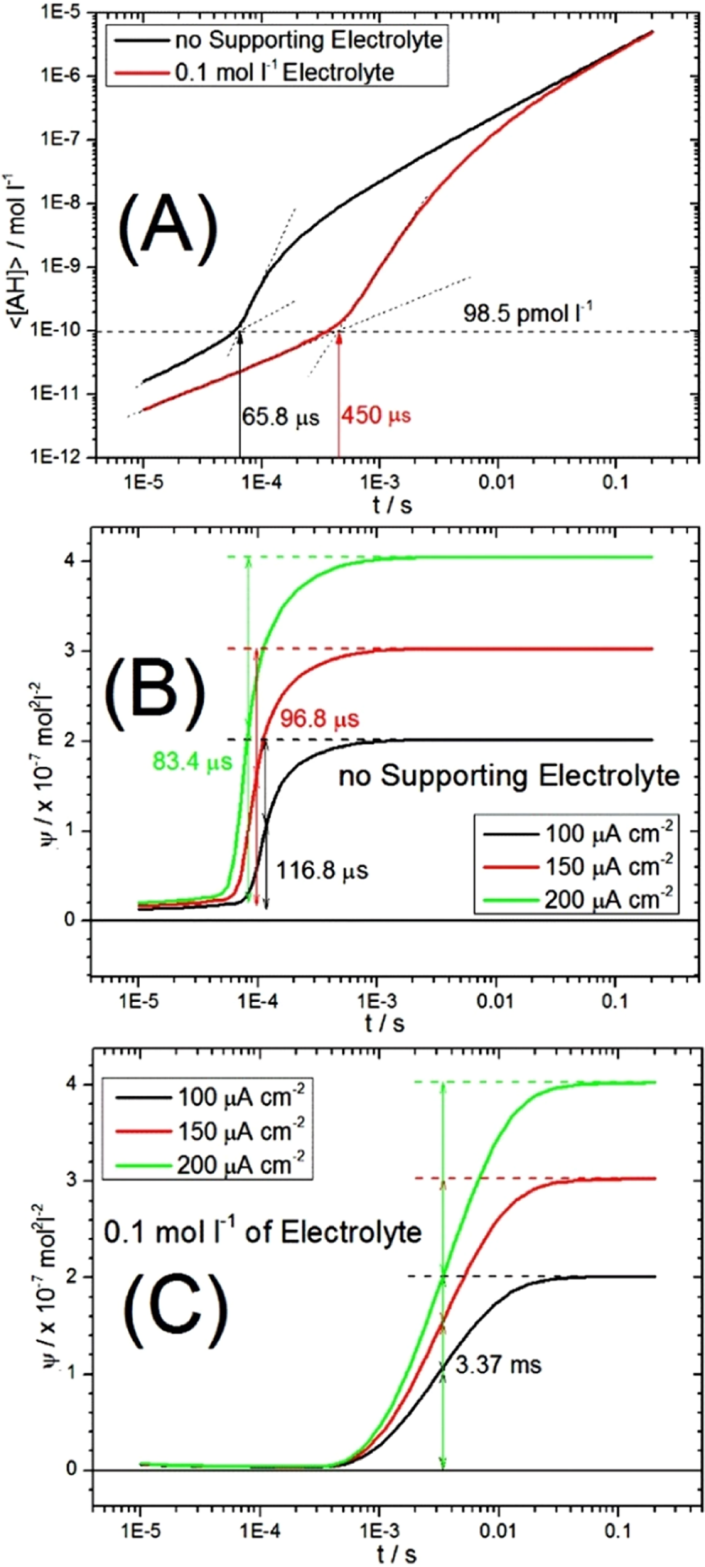
(A) Plot of the formation of AH (integrated over space and divided by space) as a function of time for (black) no supporting electrolyte and (red) with a supporting electrolyte at a constant current density of 200 μA cm^–2^. (B) Plot of Ψ = [A^–^][H^+^] (integrated over space and divided by space) as a measure of overall AH formation (see eq 24) as a function of time (no supporting electrolyte) for three current densities. (C) As before but with a supporting electrolyte.

The finite element simulation shows that the formation of AH is much faster in the absence of a supporting electrolyte. This can be rationalized by the pathway of reactants close to the surface of the interdigitated microband array electrode into the charge annihilation zone (Figure 9). The effect of the faster transport on the charge annihilation and AH production can be further demonstrated by plotting the rate of AH production versus the time. Figure 11A shows double-logarithmic plots of the AH concentration (averaged over space) versus time. An initial slope reflects the production of AH due to traces of protons available from the solvent. At 65.8 μs in the absence of a supporting electrolyte, the reaction takes off due to interdiffusion migration of A^–^ and H^+^ in the annihilation zone. In the presence of a supporting electrolyte, this process commences only at 450 μs (i.e., charge annihilation is much slower). Plot 11B shows that the annihilation reaction rate Ψ (averaged over space; see eq 24) in the absence of an electrolyte “switches on” within 120 μs of the start of electrolysis. This is contrasted to “switching on” at 3.37 ms in the presence of an electrolyte (Figure 11C). The annihilation chemistry in the absence of a supporting electrolyte is faster by more than an order of magnitude. Note that the true electrode capacitance and, therefore, the true cell RC time constant might not be fully accounted for, but the important point here is the faster flux of charged reagents between the anode and the cathode, causing faster annihilation processes in the absence of an added electrolyte.

The implications of the faster charge annihilation process are that (i) shorter-lived intermediates under paired electrosynthesis conditions are more likely to react and form the desired coupling products, (ii) charge transport from cathode to anode (see 1,1′-ferrocenedimethanol example) is enhanced, and (iii) homogeneous reaction pathways can be modified. Charge annihilation reactions (and generally reactions involving charges) are enhanced (or modified), whereas reactions with neutral species are expected to remain unaffected.

In the case of the olefin reduction reaction, the reaction step ignored in the finite element simulation was the reaction of A^–^ with the solvent BH giving B^–^ and AH (see Figure 9A,B; and the follow-up reaction steps involving AH H atom abstraction from solvent). From isotope effect data, this reaction appears crucial to give HDTET. The radical AH is highly reactive and more likely to react quickly with solvent in reaction zone C. In the future, more cases of reactions need to be investigated by comparing conditions with/without a supporting electrolyte. Better experimental tools need to be developed to map the reaction zones. The effect of not adding a supporting electrolyte could lead to beneficial changes in reaction rates and pathways (as well as improved sustainability).

## 4 Conclusions and Outlook

It has been shown that electrosynthesis at an interdigitated platinum microband array electrode in a microreactor cell is feasible, especially in the absence of LiClO_4_ electrolyte. Analysis of voltammetric data for 1,1′-ferrocenedimethanol and tetraethylethenetetracarboxylate suggest that (i) for reversible redox systems, a generator–collector feedback current occurs, dominated by cylindrical diffusion and (ii) for chemically irreversible systems, a planar diffusion layer is formed by natural convection possibly enhanced by some electroosmotic convection.^47^ The electrolysis processes at the anode and the cathode are coupled with rapid diffusion–migration of charged products toward each other. Due to migration effects on transport, the charge annihilation reaction can be more than an order of magnitude faster in the absence of a supporting electrolyte.

## Supporting Information Available

Plots of concentrations of H^+^, of A^–^, and of B^–^ in logarithmic scale in the region around the anode and the cathode and as a function of time. Plots of the electrolyte potential distribution in the region around the anode and the cathode are shown as a function of time. The report from the COMSOL simulation output (PDF)

## References

[ref1] Kingston C.; Palkowitz M. D.; Takahira Y.; Vantourout J. C.; Peters B. K.; Kawamata Y.; Baran P. S. A Survival Guide for the ″Electro-curious”. Acc. Chem. Res. 2020, 53 (1), 72–83. 10.1021/acs.accounts.9b00539. 31823612 PMC6996934

[ref2] Yan M.; Kawamata Y.; Baran P. S. Synthetic Organic Electrochemistry: Calling All Engineers. Angew. Chem., Int. Ed. 2018, 57 (16), 4149–4155. 10.1002/anie.201707584. PMC5823775 28834012

[ref3] Frontana-Uribe B. A.; Little R. D.; Ibanez J. G.; Palma A.; Vasquez-Medrano R. Organic Electrosynthesis: A Promising Green Methodology in Organic Chemistry. Green Chem. 2010, 12 (12), 2099–2119. 10.1039/c0gc00382d.

[ref4] R dos Santos T.; Harnisch F.; Nilges P.; Schröder U. Electrochemistry for Biofuel Generation: Transformation of Fatty Acids and Triglycerides to Diesel-Like Olefin/Ether Mixtures and Olefins. ChemSusChem 2015, 8 (5), 886–893. 10.1002/cssc.201403249. 25648972

[ref5] Watts K.; Baker A.; Wirth T. Microfluid Electrochemical Synthesis in Microreactors. J. Flow Chem. 2015, 4 (1), 2–11. 10.1556/JFC-D-13-00030.

[ref6] Atobe M.; Tateno H.; Matsumura Y. Fuel Cell Applications of Flow Microreactors in Electrosynthetic Processes. Chem. Rev. 2018, 118 (9), 4541–4572. 10.1021/acs.chemrev.7b00353. 28885826

[ref7] Marken F.; Wadhawan J. D. Multiphase Methods in Organic Electrosynthesis. Acc. Chem. Res. 2019, 52 (12), 3325–3338. 10.1021/acs.accounts.9b00480. 31762259

[ref8] Rodrigues H. D.; Lira J. O. D.; Padoin N.; Soares C.; Qurashi A.; Ahmed N. Ultrasound Sonoelectrochemistry: Ultrasound-assisted Organic Electrosynthesis. ACS Sustainable Chem. Eng. 2021, 9 (29), 9590–9603. 10.1021/acssuschemeng.1c02989.

[ref9] Marken F.; Sur U. K.; Coles B. A.; Compton R. G. Focused Microwaves in Electrochemical Processes. Electrochim. Acta 2006, 51 (11), 2195–2203. 10.1016/j.electacta.2005.04.081.

[ref10] Zhang S.; Findlater M. Paired Progress in Convergent Paired Electrolysis. Chem. - Eur. J. 2022, 28 (47), e202201152 10.1002/chem.202201152. 35673971

[ref11] Mo Y. M.; Lu Z. H.; Rughoobur G.; Patil P.; Gershenfeld N.; Akinwande A. L.; Buchwald S. L.; Jensen K. F. Microfluidic Electrochemistry for Single-electron Transfer Redox-neutral Reactions. Science 2020, 368, 1352–1356. 10.1126/science.aba3823. 32554592

[ref12] Mo Y. M.; Rughoobur G.; Nambiar A. M. K.; Zhang K.; Jensen K. F. A Multifunctional Microfluidic Platform for High-Throughput Experimentation of Electroorganic Chemistry. Angew. Chem., Int. Ed. 2020, 59, 20890–20894. 10.1002/anie.202009819. 32767545

[ref13] Zahrt A. F.; Mo Y. M.; Nandiwale K. Y.; Shprints R.; Heid E.; Jensen K. F. Machine-Learning-Guided Discovery of Electrochemical Reactions. J. Am. Chem. Soc. 2022, 144 (49), 22599–22610. 10.1021/jacs.2c08997. 36459170 PMC9756344

[ref14] Belmont C.; Girault H. H. Coplanar Interdigitated Band Electrodes for Synthesis 1. Ohmic Loss Evaluation. J. Appl. Electrochem. 1994, 24 (6), 475–480. 10.1007/BF00249845.

[ref15] Belmont C.; Girault H. H. Coplanar Interdigitated Band Electrodes for Electrosynthesis 2. Methoxylation of Furan. J. Appl. Electrochem. 1994, 24 (8), 719–724. 10.1007/BF00578085.

[ref16] Mengeaud V.; Bagel O.; Ferrigno R.; Girault H. H.; Haider A. A Ceramic Electrochemical Microreactor for the Methoxylation of Methyl-2-furoate with Direct Mass Spectrometry Coupling. Lab Chip 2002, 2 (1), 39–44. 10.1039/b109587k. 15100860

[ref17] Ferrigno R.; Comninellis C.; Reid V.; Modes C.; Scannell R.; Girault H. H. Coplanar Interdigitated Band Electrodes for Electrosynthesis. 6. Hypochlorite Electrogeneration from Sea Water Electrolysis. Electrochim. Acta 1999, 44 (17), 2871–2878. 10.1016/S0013-4686(99)00005-5.

[ref18] Belmont C.; Girault H. H. Coplanar Interdigitated Band Electrodes for Electrosynthesis. 3. Epoxidaton of Propylene. Electrochim. Acta 1995, 40 (15), 2505–2510. 10.1016/0013-4686(95)00133-Y.

[ref19] Ferrigno R.; Josserand J.; Brevet P. F.; Girault H. H. Coplanar Interdigitated Band Electrodes for Electrosynthesis. Part 5: Finite Element Simulation of Paired Reactions. Electrochim. Acta 1998, 44 (4), 587–595. 10.1016/S0013-4686(98)00187-X.

[ref20] Matsue T. Electrochemical Sensors Using Microarray Electrodes. TrAC, Trends Anal. Chem. 1993, 12 (3), 100–108. 10.1016/0165-9936(93)88009-T.

[ref21] Barnes E. O.; Lewis G. E. M.; Dale S. E. C.; Marken F.; Compton R. G. Generator-Collector Double Electrode Systems: A Review. Analyst 2012, 137 (5), 1068–1081. 10.1039/c2an16174e. 22274834

[ref22] Suzuki H. Advances in the Microfabrication of Electrochemical Sensors and Systems. Electroanalysis 2000, 12 (9), 703–715. 10.1002/1521-4109(200005)12:9<703::AID-ELAN703>3.0.CO;2-7.

[ref23] Fiaccabrino G. C.; Tang X. M.; Skinner N.; deRooij N. F.; KoudelkaHep M. Electrochemical Characterization of Thin-Film Carbon Interdigitated Electrode Arrays. Anal. Chim. Acta 1996, 326 (1–3), 155–161. 10.1016/0003-2670(96)00068-2.

[ref24] Zhang W.; Xie S. A.; Li M.; Chen H. J.; Ma L.; Jia J. P. Electrochemical Characteristics of an Interdigitated Microband Electrode Array of Boron-Doped Diamond Film. Collect. Czech. Chem. Commun. 2009, 74 (3), 393–407. 10.1135/cccc2008161.

[ref25] Ueno K.; Hayashida M.; Ye J. Y.; Misawa H. Fabrication and Electrochemical Characterization of Interdigitated Nanoelectrode Arrays. Electrochem. Commun. 2005, 7 (2), 161–165. 10.1016/j.elecom.2004.12.002.

[ref26] Li W.; Nonaka T.; Chou T. C. Paired Electrosynthesis of Organic Compounds. Electrochemistry 1999, 67 (1), 4–10. 10.5796/electrochemistry.67.4.

[ref27] Wu T.; Moeller K. D. Paired Electrolysis. In Electrochemistry in Organic Synthesis; Ackermann L., Ed.; 2022; pp 481–512.

[ref28] Zhang W. Z.; Hong N. M.; Song L.; Fu N. K. Reaching the Full Potential of Electroorganic Synthesis by Paired Electrolysis. Chem. Record 2021, 21 (9), 2574–2584. 10.1002/tcr.202100025. 33835697

[ref29] Marken F.; Cresswell A. J.; Bull S. D. Recent Advances in Paired Electrosynthesis. Chem. Record 2021, 21 (9), 2585–2600. 10.1002/tcr.202100047. 33834595

[ref30] Paddon C. A.; Atobe M.; Fuchigami T.; He P.; Watts P.; Haswell S. J.; Pritchard G. J.; Bull S. D.; Marken F. Towards Paired and Coupled Electrode Reactions for Clean Organic Microreactor Electrosyntheses. J. Appl. Electrochem. 2006, 36 (6), 617–634. 10.1007/s10800-006-9122-2.

[ref31] Amemiya F.; Horii D.; Fuchigami T.; Atobe M. Self-supported Paired Electrosynthesis using a Microflow Reactor without Intentionally Added Electrolyte. J. Electrochem. Soc. 2008, 155 (11), E162–E165. 10.1149/1.2975823.

[ref32] Bard A. J.; Faulkner L. R. Electrochemical Methods, 2nd ed.; John Wiley, 2001; p 140.

[ref33] Marken F.; Goldfarb D. L.; Compton R. G. Sonoelectrochemistry in highly resistive media: Mass transport effects. Electroanalysis 1998, 10 (8), 562–566. 10.1002/(SICI)1521-4109(199807)10:8<562::AID-ELAN562>3.0.CO;2-7.

[ref34] Lide D. R. CRC Handbook of Chemistry and Physics, Internet Version 200585th ed.; CRC Press: Boca Raton, FL, 2005; Vol. 85. http://www.hbcpnetbase.com.

[ref35] Streeter I.; Compton R. G. Numerical Simulation of Potential Step Chronoamperometry at Low Concentrations of Supporting Electrolyte. J. Phys. Chem. C 2008, 112 (35), 13716–13728. 10.1021/jp804442m.

[ref36] Liu Y.; Zhang Q.; Chen S. The Voltammetric Responses of Nanometer-sized Electrodes in Weakly Supported Electrolyte: A Theoretical Study. Electrochim. Acta 2010, 55 (27), 8280–8286. 10.1016/j.electacta.2010.04.028.

[ref37] Tang Y.; Dubbeldam D.; Tanase S. Water-Ethanol and Methanol-Ethanol Separations Using in Situ Confined Polymer Chains in a Metal-Organic Framework. ACS Appl. Mater. Interfaces 2019, 11 (44), 41383–41393. 10.1021/acsami.9b14367. 31600050 PMC6838788

[ref38] Solntsev K. M.; Huppert D.; Agmon N.; Tolbert L. M. Photochemistry of “Super” Photoacids. 2. Excited-State Proton Transfer in Methanol/Water Mixtures. J. Phys. Chem. A 2000, 104 (19), 4658–4669. 10.1021/jp994454y.

[ref39] Hawlicka E. Self-Diffusion of Sodium, Chloride and Iodide Ions in Methanol-Water Mixture. Z. Naturforsch. A 1986, 41 (7), 939–943. 10.1515/zna-1986-0707.

[ref40] Mohsen-Nia M.; Amiri H.; Jazi B. Dielectric Constants of Water, Methanol, Ethanol, Butanol and Acetone: Measurement and Computational Study. J. Solution Chem. 2010, 39 (5), 701–708. 10.1007/s10953-010-9538-5.

[ref41] Stott S. J.; Mortimer R. J.; McKenzie K. J.; Marken F. Mesoporous TiO_2_ Carboxymethyl-γ-cyclodextrate Multi-layer Host Films: Effects on Adsorption and Electrochemistry of 1,1′-Ferrocenedimethanol. Analyst 2005, 130 (3), 358–363. 10.1039/B412698J.

[ref42] Aoki K. Theory of Ultramicroelectrodes. Electroanalysis 1993, 5 (8), 627–639. 10.1002/elan.1140050802.

[ref43] Nowicka A. M.; Donten M.; Palys M.; Van den Bossche B.; Stojek Z. Voltammetric Studies of Parallel Electrode Processes under Low Ionic Strength Conditions. Influence of Convection. Electroanalysis 2006, 18 (7), 641–648. 10.1002/elan.200503452.

[ref44] Atkins P. W.; de Paula J. Physical Chemistry, 8th ed.; Oxford University Press, 2006; p 776.

[ref45] Bard A. J.; Faulkner L. R. Electrochemical Methods, 2nd ed.; John Wiley, 2001; p 231.

[ref46] Caban K.; Kudelski A.; Donten M.; Stojek Z. Voltammetry of Undiluted Redox Systems Backed by in-situ Raman Spectroscopy. Evidence for Strong Accumulation of Ions in the Diffusion Layer at Microelectrode Surface. Electrochem. Commun. 2003, 5 (5), 412–415. 10.1016/S1388-2481(03)00091-2.

[ref47] Caban K.; Hyk N.; Donten M.; Stojek Z. Influence of Gravitation on Steady-state Currents of Undiluted Alcohols at Microelectrodes. Chem. Anal. 2001, 46 (6), 813–822.

