# Supplementary material for: Paired Electrosynthesis at Interdigitated Microband Electrodes: Exploring Diffusion and Reaction Zones in the Absence of a Supporting Electrolyte
Source: ACS Meas Sci Au. 2024 Apr 17;4(3):294–306. doi: 10.1021/acsmeasuresciau.4c00009 (PMC11191726; doi:10.1021/acsmeasuresciau.4c00009)
Supplement: Supplementary file 1 — tg4c00009_si_001.pdf [file tg4c00009_si_001.pdf]

Supporting Information

Paired Electrosynthesis at Interdigitated Microband Electrodes: Exploring Diffusion and Reaction Zones in the Absence of Supporting Electrolyte

Tingran Liu [1], Evaldo Batista Carneiro-Neto [1,2], Ernesto Pereira [2], James E. Taylor [1], Philip J. Fletcher [3], and Frank Marken*[1]

[1] *Department of Chemistry, University of Bath, Claverton Down, Bath BA2 7AY, UK*

[2] *Department of Chemistry, Federal University of São Carlos, Rod. Washington Luiz, Km 235, CEP 13565-905, São Carlos, SP, Brazil*

[3] *University of Bath, Materials & Chemical Characterisation Facility, MC^2, Bath BA2 7AY, UK*

Contents

1. Figure S1. Plots of concentration of H^+, in logarithmic scale, in the region around anode and cathode and as a function of time ………………………………………….. 3

2. Figure S2. Plots of concentration of A^-, in logarithmic scale, in the region around anode and cathode and as a function of time. ………………………………………….. 4

3. Figure S3. Plots of concentration of B^-, in logarithmic scale, in the region around anode and cathode and as a function of time. …………………………………………. 5

4. Figure S4. Plots of electrolyte potential in the region around anode and cathode and as a function of time. ……………………………………………………………….. 6

5. Report from COMSOL Simulation. ……………………………………………… 7

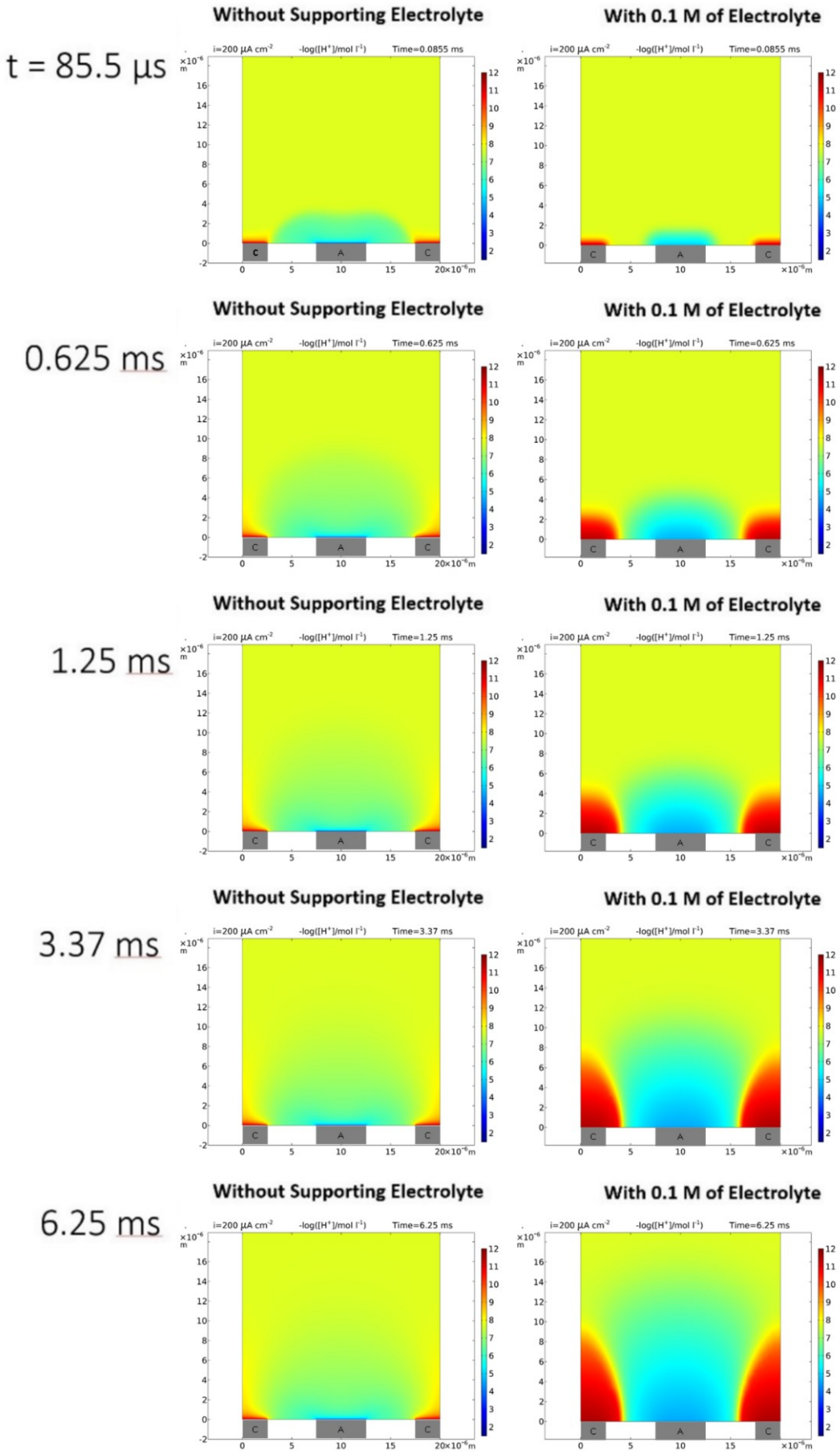

Figure S1. Plots of concentration of H^+, in logarithmic scale, in the region around anode and cathode and as a function of time. The left y-axis gives the distance (in µm) from the electrode surface and the right y-axis shows the colour coding in terms of the negative decadic exponent for concentration (*i.e.* dark red = 10^{-12} mol dm^{-3} and dark blue = 10^{-2} mol dm^{-3}).

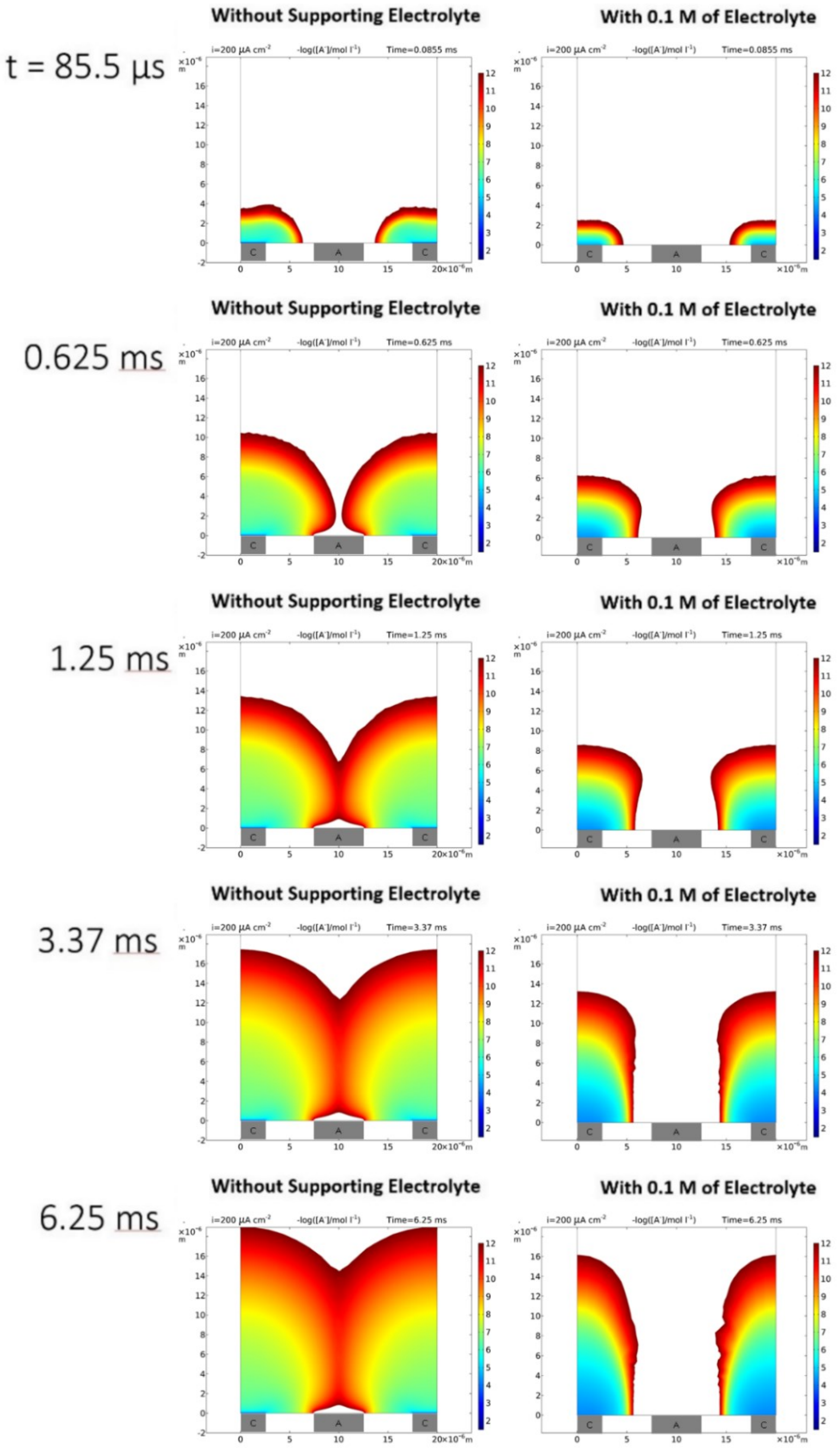

Figure S2. Plots of concentration of A^{-}, in logarithmic scale, in the region around anode and cathode and as a function of time. The left y-axis gives the distance (in µm) from the electrode surface and the right y-axis shows the colour coding in terms of the negative decadic exponent for concentration (*i.e.* dark red = 10^{-12} mol dm^{-3} and dark blue = 10^{-2} mol dm^{-3}).

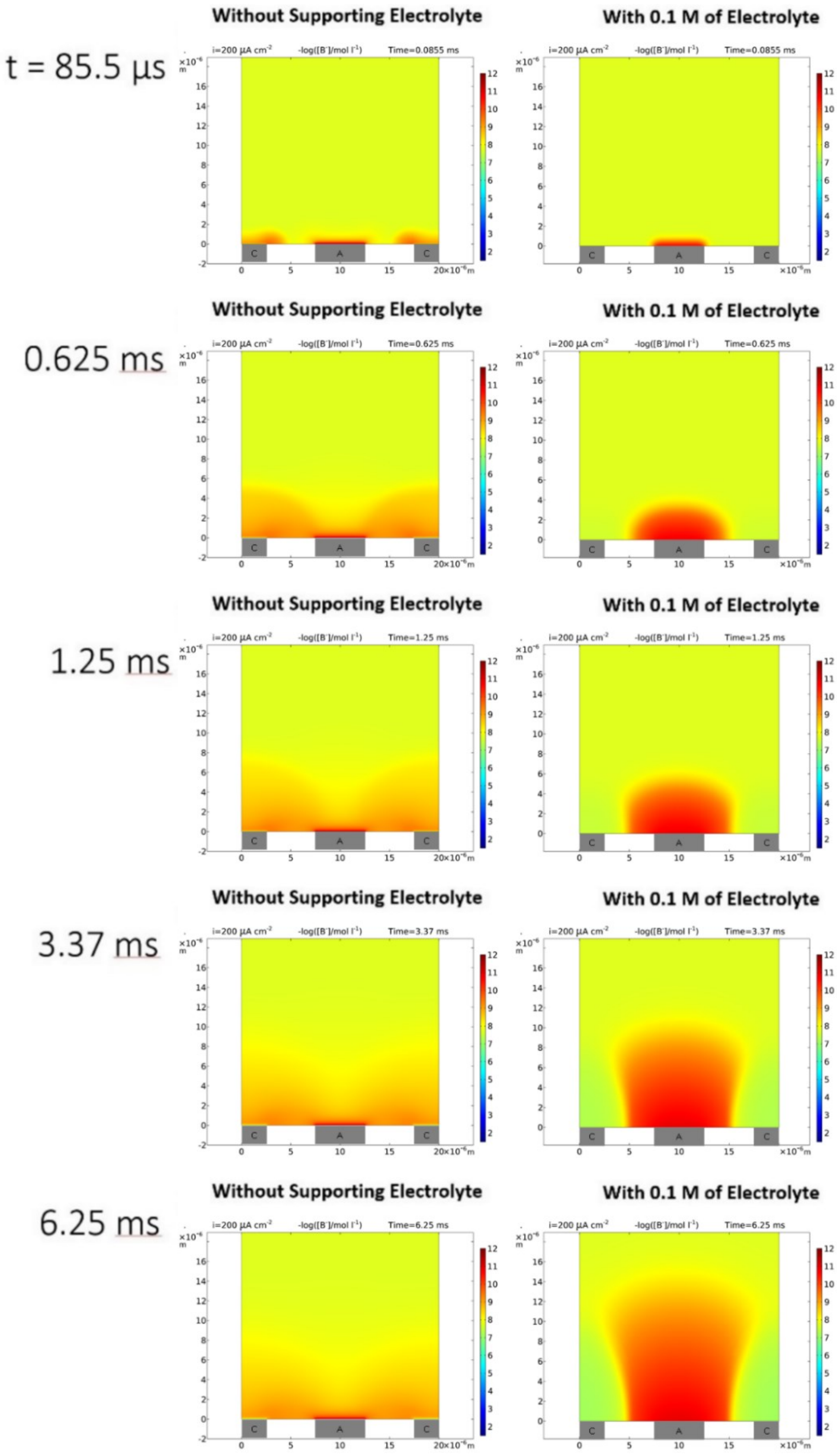

Figure S3. Plots of concentration of B^-, in logarithmic scale, in the region around anode and cathode and as a function of time. The left y-axis gives the distance (in µm) from the electrode surface and the right y-axis shows the colour coding in terms of the negative decadic exponent for concentration (*i.e.* dark red = 10^{-12} mol dm^{-3} and dark blue = 10^{-2} mol dm^{-3}).

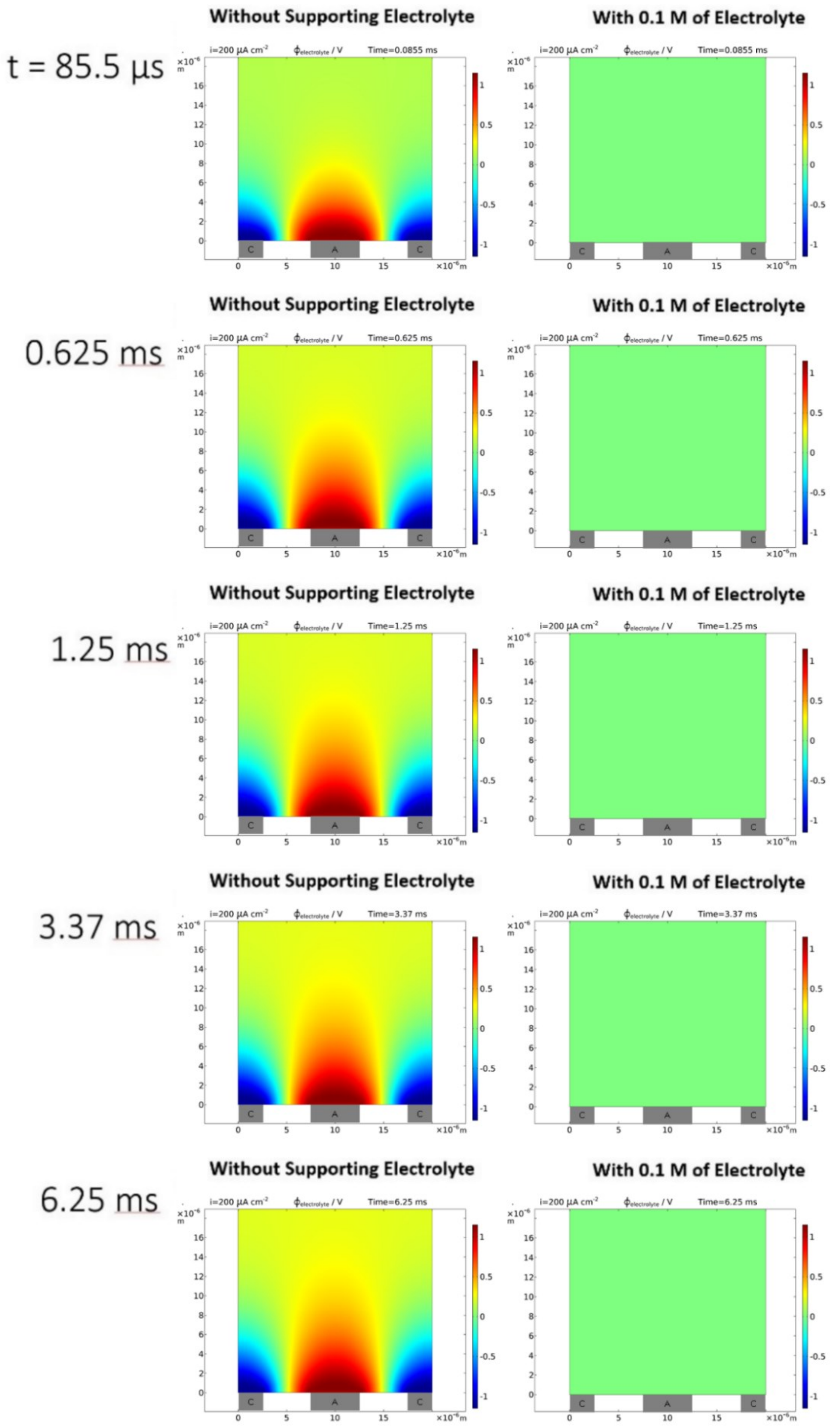

Figure S4. Plots of electrolyte potential in the region around anode and cathode and as a function of time. The left y-axis gives the distance (in µm) from the electrode surface and the right y-axis shows the colour coding in terms of the potential (*i.e.* dark red = 1V and dark blue -1 V).

Report from COMSOL Simulation

M6T 2023 10 12

Report date

Mar 19, 2024, 9:45:22 AM

Contents

1. Global Definitions 9
1.1. Parameters 9
2. Component 1 12
2.1. Definitions 12
2.2. Geometry 1 16
2.3. Tertiary Current Distribution, Nernst-Planck 17
2.4. Mesh 1 18
3. Study 1 19
3.1. Parametric Sweep 19
3.2. Current Distribution Initialization 19
3.3. Time Dependent 20
4. Results 22
4.1. Datasets 22
4.2. Plot Groups 25

1 Global Definitions

Date	Mar 18, 2024, 11:37:32 AM

GLOBAL SETTINGS

Name	M6T 2023 10 12.mph
Path	/home/liec-eletroquimica/Documentos/Evaldo/model_ref/M6T_2023_10_12.mph
Version	COMSOL Multiphysics 6.0 (Build: 354)

USED PRODUCTS

Electrochemistry Module
COMSOL Multiphysics

COMPUTER INFORMATION

CPU	Intel(R) Core(TM) i7-7800X CPU @ 3.50GHz, 6 cores
Operating system	Linux

1.1 PARAMETERS

1.1.1 Parameters 1

PARAMETERS 1

Name	Expression	Value	Description
tau	0.2[s]	0.2 s	Total time
alpha_ox	0.5	0.5	Oxidation reaction transfer coefficient
eps_MetOH	33	33	Dielectric constant of methanol
r_MetOH	0.36[nm]/2	1.8E−10 m	Kinetic diameter of the methanol molecule
mu	(2 + sqrt(3))*r_MetOH	6.7177E−10 m	Thickness of the Stern layer
C_ref	1[mol/l]	1000 mol/m³	Reference concentration
alpha_red	0.5	0.5	Reduction reaction transfer coefficient
eps_S	11	11	Dielectric constant inside the Stern layer
C_S	epsilon0_const*eps_S/mu	0.14498 F/m²	Capacitance of the Stern layer

Name	Expression	Value	Description
D_H	2.34e-5[cm^2/s]	2.34E−9 m^2/s	Diffusion coefficient of H^+ in methanol
D_Dn	1.378e-9[m^2/s]	1.378E−9 m^2/s	Diffusion coefficient of D^- in methanol
D_Cp	1.098e-9[m^2/s]	1.098E−9 m^2/s	Diffusion coefficient of C^+ in methanol
D_An	1e-9[m^2/s]	1E−9 m^2/s	Diffusion coefficient of A^- in methanol
D_Bn	1e-9[m^2/s]	1E−9 m^2/s	Diffusion coefficient of B^- in methanol
D_AH	1e-9[m^2/s]	1E−9 m^2/s	Diffusion coefficient of AH in methanol
phiext_anode	0[V]	0 V	External electric potential at the anode
phiext_cathode	0[V]	0 V	External electric potential at the cathode

1.1.2 Geometric Parameters

GEOMETRIC PARAMETERS

Name	Expression	Value	Description
h_dom	8*sqrt(pi*D_AH*tau)	2.0053E−4 m	Height of the domain
w_gap	5[um]	5E−6 m	Size of the gap between the electrodes
L_electrode	6[mm]	0.006 m	Length of the electrodes
w_electrode	5[um]	5E−6 m	Width of the electrodes
w_dom	2*w_gap + 2*w_electrode	2E−5 m	Width of the domain

1.1.3 Kinetic Parameters

KINETIC PARAMETERS

Name	Expression	Value	Description
i0_ox	1.0e2[mA/cm^2]	1000 A/m^2	Exchange current density of oxidation reaction
i0_red	1.0e2[mA/cm^2]	1000 A/m^2	Reduction reaction transfer coefficient

Name	Expression	Value	Description
kab	4e7[m^3/(mol*s)]	4E7 m³/(s·mol)	rate constant for the alcohol association reaction
k3	8e7[m^3/(mol*s)]	8E7 m³/(s·mol)	rate cosntant for the formation of AH

1.1.4 Equilibrium Parameters

EQUILIBRIUM PARAMETERS

Name	Expression	Value	Description
Ka	10^ - 15.5	3.1623E−16	Alcohol equilibrium constant
cH_ini	(cAn_ini + sqrt(cAn_ini^2 + 4*Ka*C_ref^2))/2	1.7783E−5 mol/m³	Initial concentration of H^+
cBn_ini	Ka*C_ref^2/cH_ini	1.7782E−5 mol/m³	Initial concentration of B^-
cAn_ini	1e-12[mol/l]	1E−9 mol/m³	Initial concentration of A^-
cAH_ini	0[mol/l]	0 mol/m³	Initial concentration of AH
C_sup	0.1[mol/l]	100 mol/m³	Initial concentration of supporting electrolyte

2 Component 1

2.1 DEFINITIONS

2.1.1 Variables

Variables 1

SELECTION

Geometric entity level	Entire model

Name	Expression	Unit	Description
deltaphi	tcd.phisext - phil	V	Electrode-OHP potential difference
rho_surf	C_S*deltaphi	C/m^2	Surface charge density

2.1.2 Nonlocal Couplings

Average 1

Coupling type	Average
Operator name	aveop_dom

SELECTION

Geometric entity level	Domain
Selection	Geometry geom1: Dimension 2: Domain 1

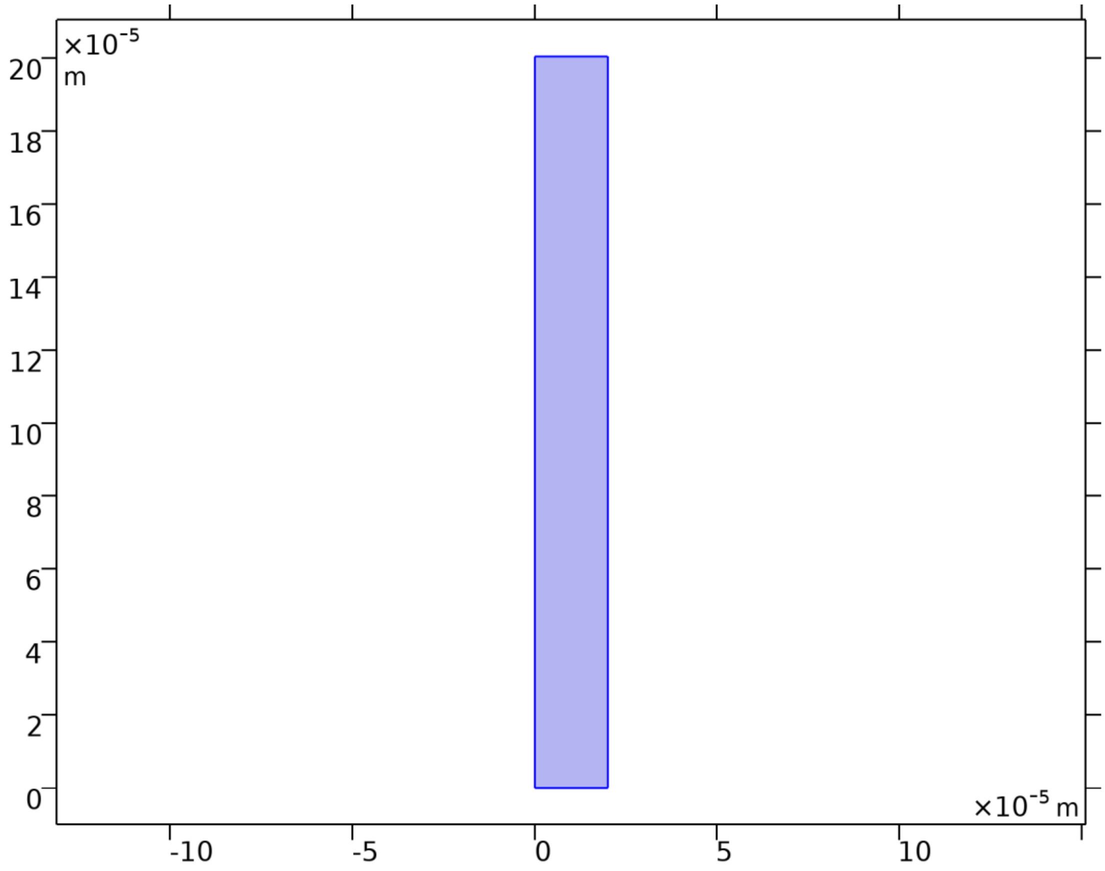

Selection

Average 2

Coupling type	Average
Operator name	aveop_anode

SELECTION

Geometric entity level	Boundary
Selection	Geometry geom1: Dimension 1: Boundary 5

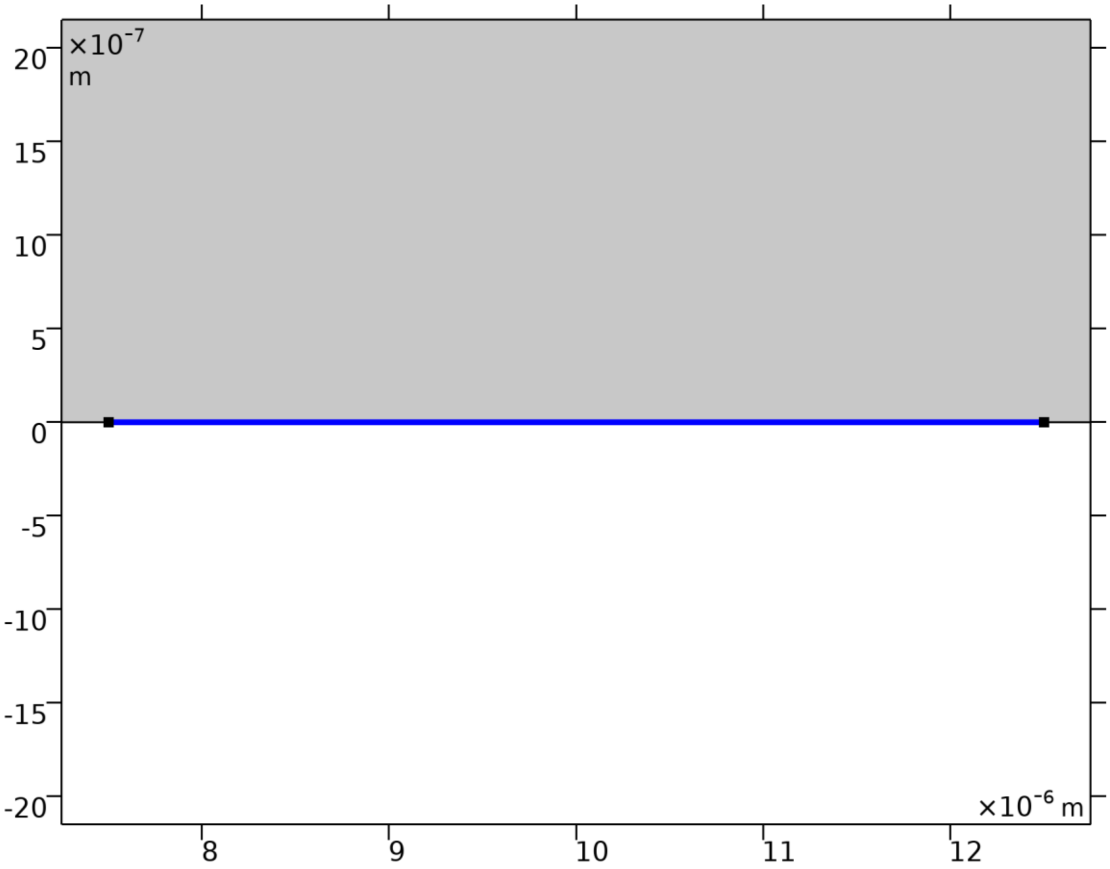

Selection

Average 3

Coupling type	Average
Operator name	aveop_cathode

SELECTION

Geometric entity level	Boundary
Selection	Geometry geom1: Dimension 1: Boundaries 2, 7

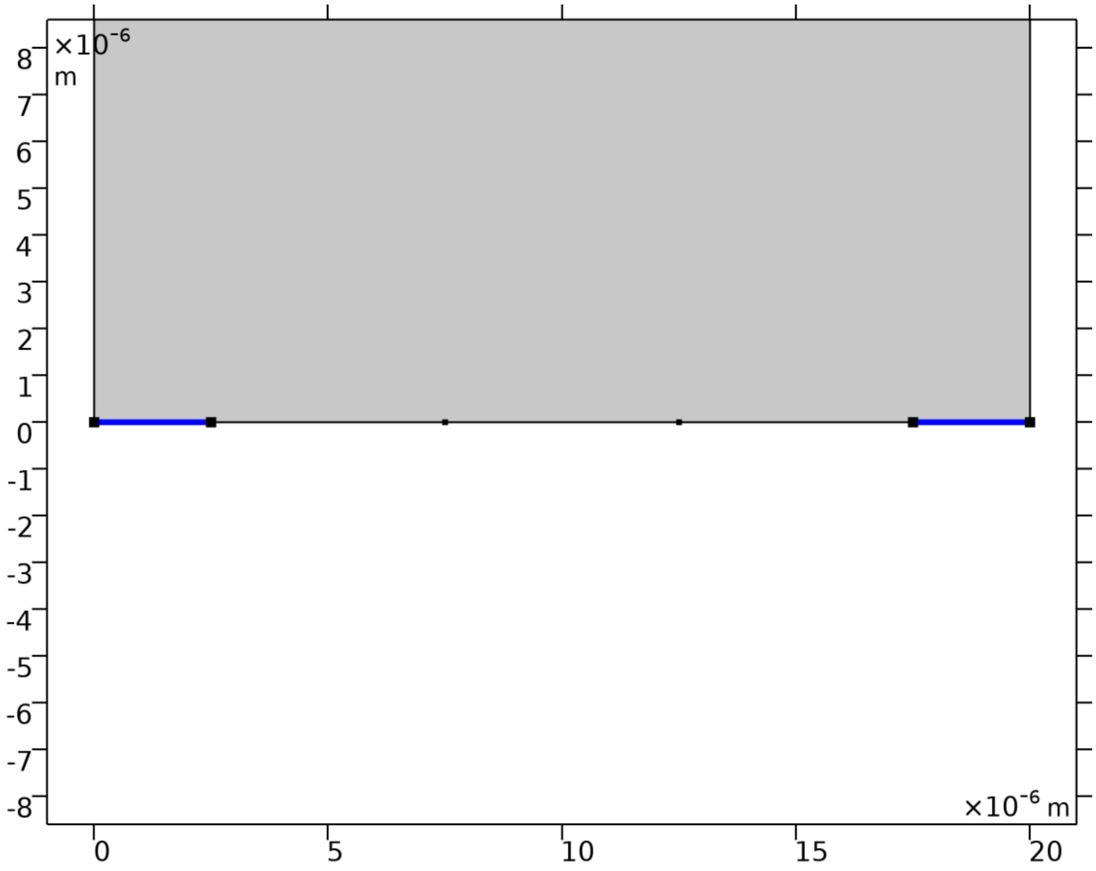

Selection

2.1.3 Coordinate Systems

Boundary System 1

Coordinate system type	Boundary system
Tag	sys1

COORDINATE NAMES

First	Second	Third
t1	n	to

2.2 GEOMETRY 1

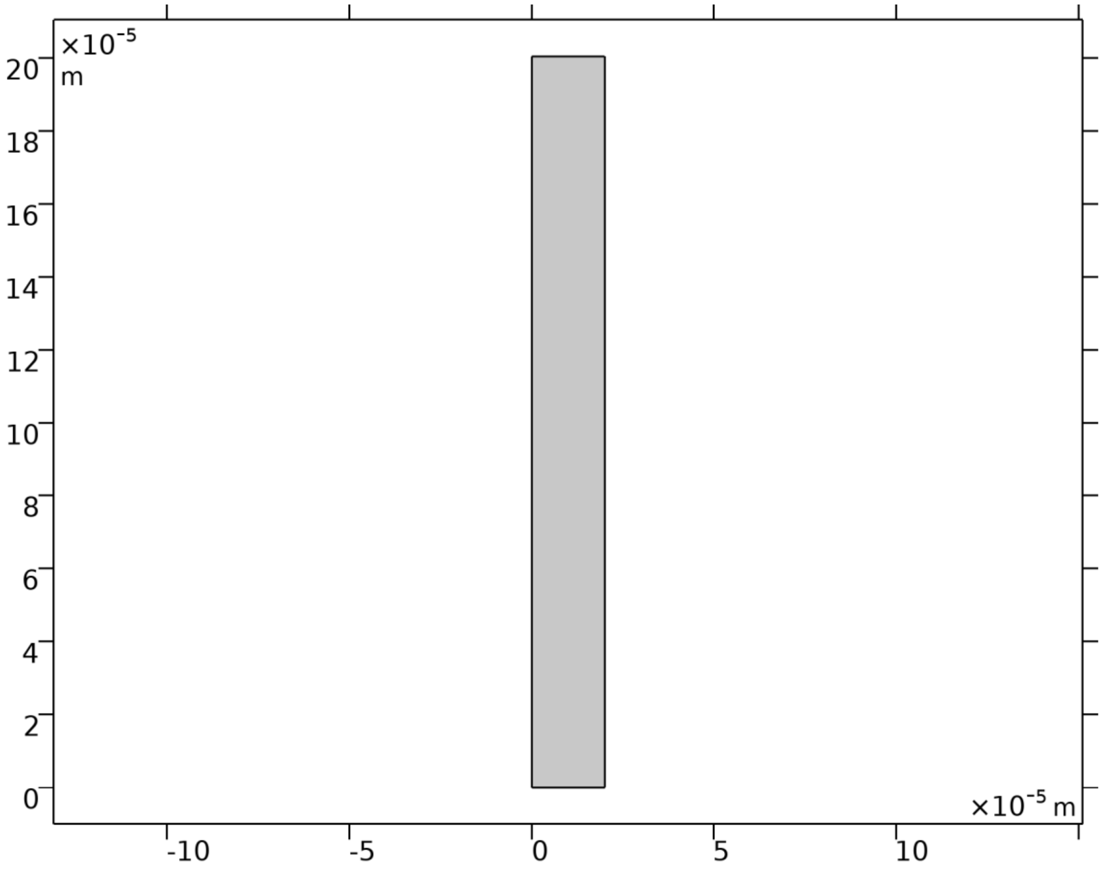

Geometry 1

UNITS

Length unit	m
Angular unit	deg

2.3 TERTIARY CURRENT DISTRIBUTION, NERNST-PLANCK

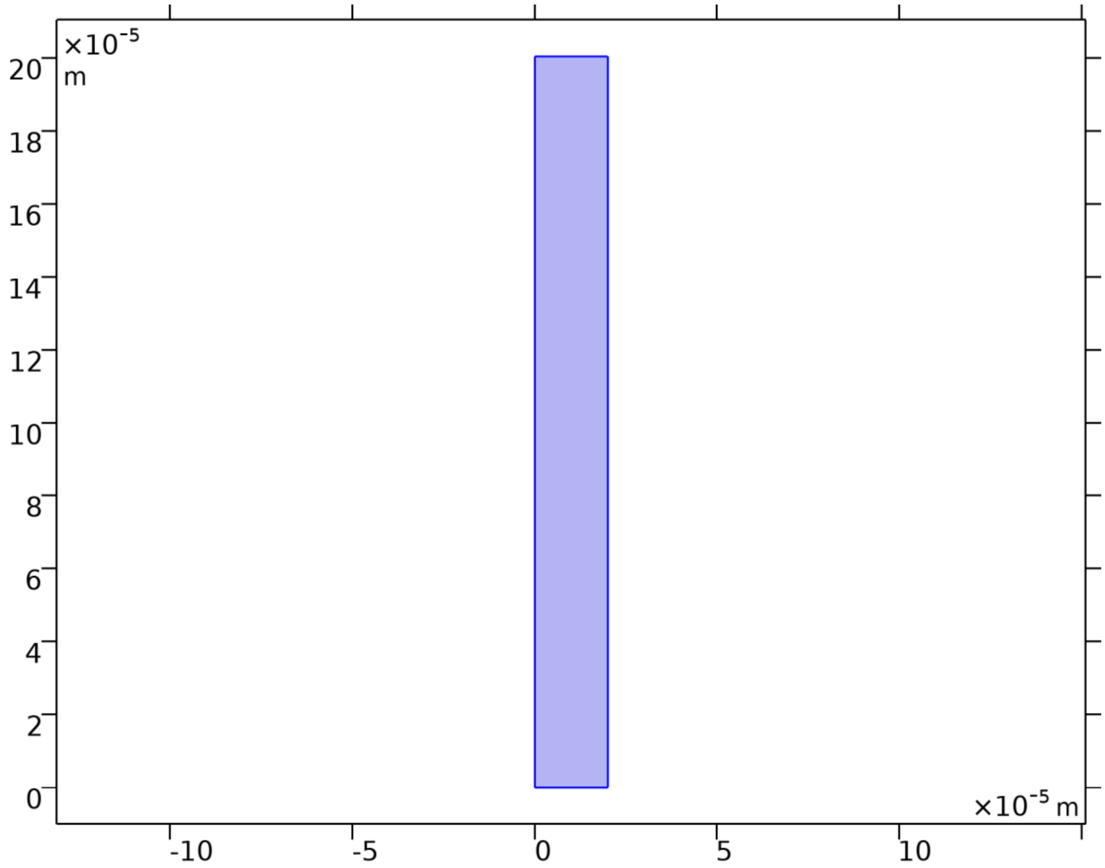

Tertiary Current Distribution, Nernst-Planck

EQUATIONS

$$\nabla \cdot \mathbf{J}_i + \mathbf{u} \cdot \nabla c_i = R_i$$
$$\nabla \cdot \mathbf{D}_\mathrm{l} = F \sum_i z_i c_i$$
$$\nabla \cdot \mathbf{i}_\mathrm{s} = Q_\mathrm{s}$$
$$\mathbf{J}_i = -D_i \nabla c_i - z_i u_{\mathrm{m},i} F c_i \nabla \phi_\mathrm{l}$$
$$\mathbf{D}_\mathrm{l} = -\epsilon_0 \epsilon_\mathrm{r} \nabla \phi_\mathrm{l}$$
$$\mathbf{i}_\mathrm{s} = -\sigma_\mathrm{s} \nabla \phi_\mathrm{s}$$
$$\phi_\mathrm{l} = \mathrm{phil}, \quad \phi_\mathrm{s} = \mathrm{phis}$$

FEATURES

Name	Level
Electrolyte 1	Domain
No Flux 1	Boundary
Insulation 1	Boundary
Initial Values 1	Domain

Name	Level
Electrode Surface 1	Boundary
Electrode Surface 2	Boundary
Surface Charge Density 1	Boundary
Electrolyte Potential 1	Point
Reactions 1	Domain

2.4 MESH 1

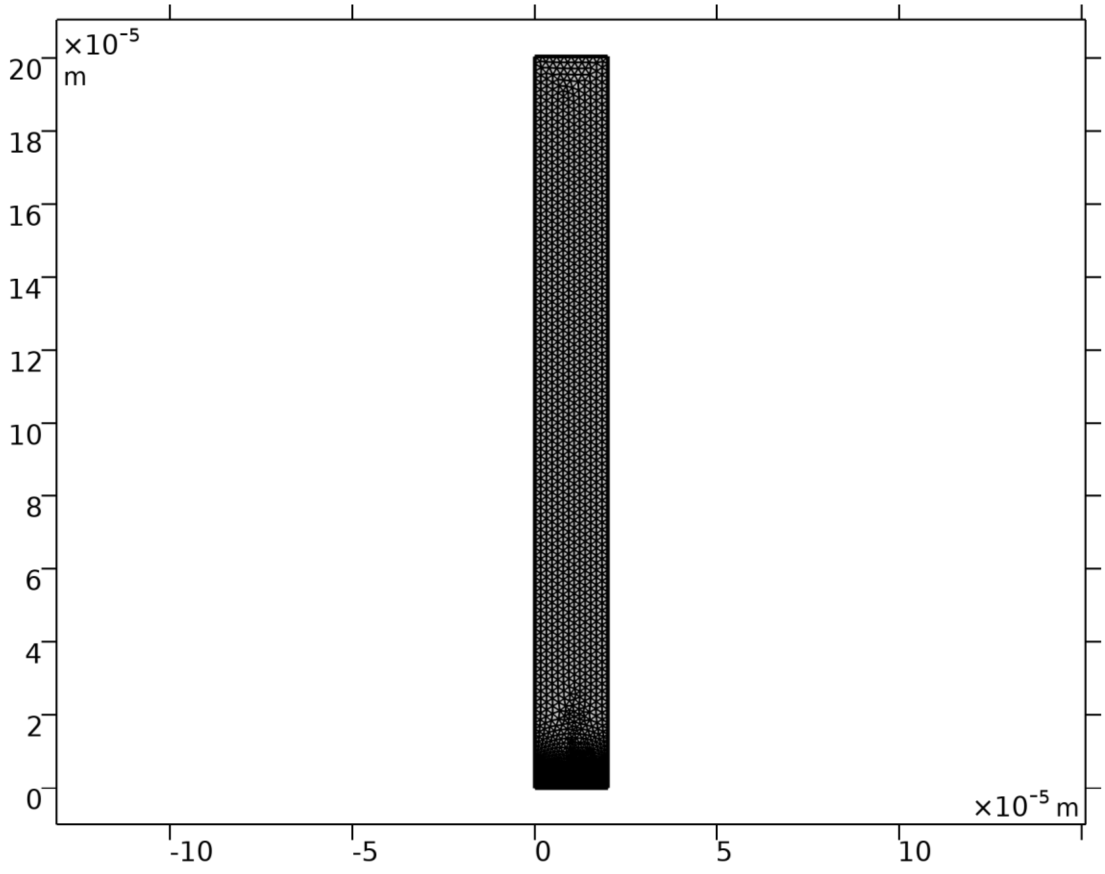

Mesh 1

3 Study 1

COMPUTATION INFORMATION

Computation time	2 h 12 min 33 s

3.1 PARAMETRIC SWEEP

Parameter name	Parameter value list	Parameter unit
phiext_anode	-0.61365	V
phiext_cathode	0.54774	V

STUDY SETTINGS

Description	Value
Sweep type	Specified combinations
Parameter name	{phiext_anode, phiext_cathode}
Unit	{V, V}

PARAMETERS

Parameter name	Parameter value list	Parameter unit
phiext_anode (External electric potential at the anode)	-0.61365	V
phiext_cathode (External electric potential at the cathode)	0.54774	V

3.2 CURRENT DISTRIBUTION INITIALIZATION

STUDY SETTINGS

Description	Value
Include geometric nonlinearity	Off

STUDY SETTINGS

Description	Value
Current distribution type	Secondary

PHYSICS AND VARIABLES SELECTION

Physics interface	Discretization
Tertiary Current Distribution, Nernst-Planck (tcd)	physics

MESH SELECTION

Geometry	Mesh
Geometry 1 (geom1)	mesh1

3.3 TIME DEPENDENT

Times	Unit
0 10^{range(-5,0.05,log10(tau*1[1/s]))}	s

STUDY SETTINGS

Description	Value
Include geometric nonlinearity	Off

STUDY SETTINGS

Description	Value
Output times	{0, 1.0E-5, 1.122018454301963E-5, 1.2589254117941661E-5, 1.4125375446227555E-5, 1.584893192461114E-5, 1.778279410038923E-5, 1.9952623149688786E-5, 2.238721138568338E-5, 2.5118864315095822E-5, 2.818382931264455E-5, 3.1622776601683795E-5, 3.5481338923357534E-5, 3.9810717055349695E-5, 4.466835921509635E-5, 5.011872336272725E-5, 5.623413251903491E-5, 6.309573444801929E-5, 7.079457843841373E-5, 7.943282347242822E-5, 8.912509381337459E-5, 1.0E-4, 1.122018454301963E-4, 1.2589254117941674E-4, 1.4125375446227554E-4, 1.5848931924611142E-4, 1.7782794100389227E-4, 1.9952623149688788E-4, 2.23872113856834E-4, 2.511886431509582E-4, 2.818382931264455E-4, 3.1622776601683794E-4, 3.548133892335753E-4, 3.9810717055349735E-4, 4.466835921509635E-4, 5.011872336272725E-4, 5.623413251903491E-4, 6.30957344480193E-4, 7.07945784384138E-4, 7.943282347242822E-4, 8.912509381337459E-4, 0.001, 0.0011220184543019641, 0.0012589254117941675, 0.001412537544622754, 0.001584893192461114, 0.0017782794100389228, 0.0019952623149688807, 0.00223872113856834, 0.002511886431509582, 0.002818382931264455, 0.0031622776601683794, 0.0035481338923357567, 0.003981071705534973, 0.004466835921509635, 0.005011872336272725, 0.005623413251903491, 0.006309573444801936, 0.00707945784384138, 0.00794328234724282, 0.008912509381337459, 0.01, 0.011220184543019640, 0.012589254117941675, 0.014125375446227554, 0.015848931924611140, 0.017782794100389230, 0.019952623149688810, 0.022387211385683400, 0.025118864315095820, 0.028183829312644550, 0.031622776601683790, 0.035481338923357565, 0.039810717055349734, 0.044668359215096350, 0.050118723362727250, 0.056234132519034910, 0.063095734448019360, 0.070794578438413800, 0.079432823472428220, 0.089125093813374590, 0.1, 0.1122018454301963, 0.12589254117941687, 0.14125375446227553, 0.15848931924611143, 0.1778279410038923, 0.1995262314968879}

PHYSICS AND VARIABLES SELECTION

Physics interface	Discretization
Tertiary Current Distribution, Nernst-Planck (tcd)	physics

MESH SELECTION

Geometry	Mesh
Geometry 1 (geom1)	mesh1

4 Results

4.1 DATASETS

4.1.1 Study 1/Solution 1

SOLUTION

Description	Value
Solution	Solution 1
Component	Component 1 (comp1)

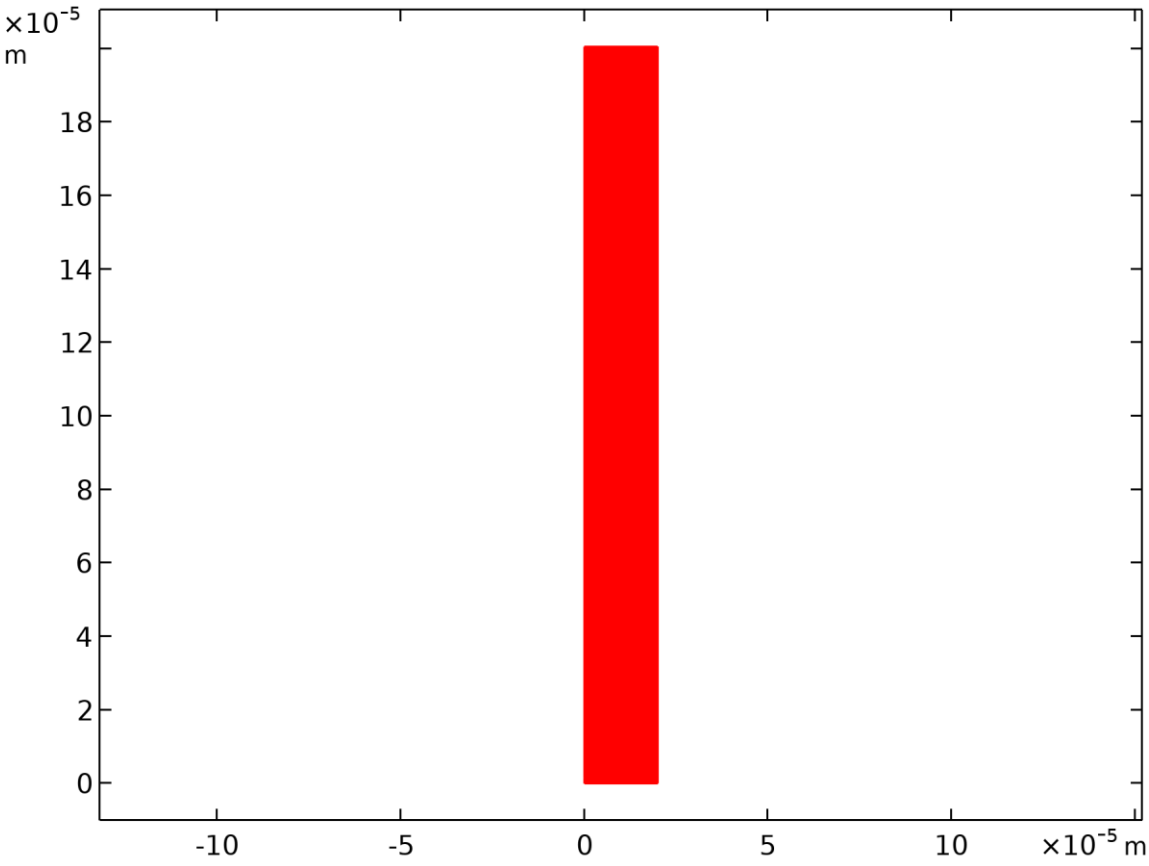

Dataset: Study 1/Solution 1

4.1.2 Study 1/Solution Store 1

SOLUTION

Description	Value
Solution	Solution Store 1
Component	Component 1 (comp1)

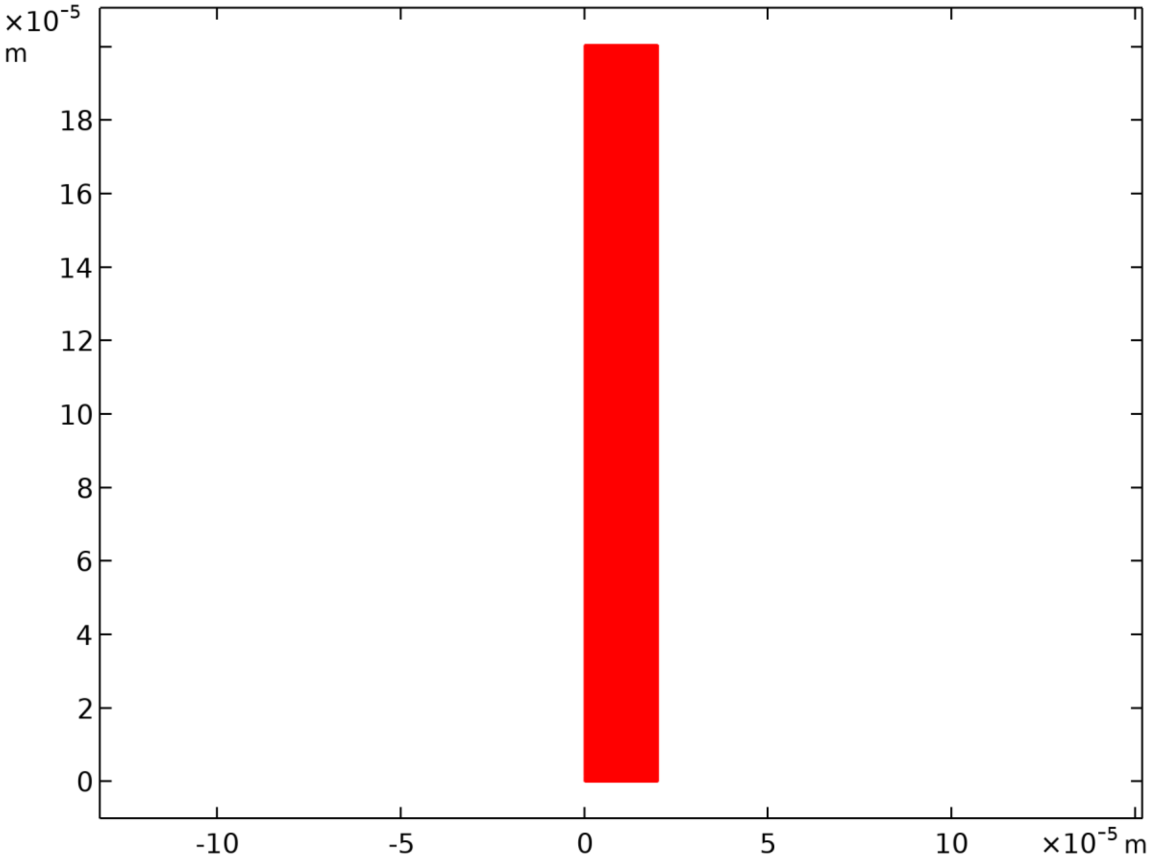

Dataset: Study 1/Solution Store 1

4.1.3 Study 1/Parametric Solutions 1

SOLUTION

Description	Value
Solution	Parametric Solutions 1
Component	Component 1 (comp1)

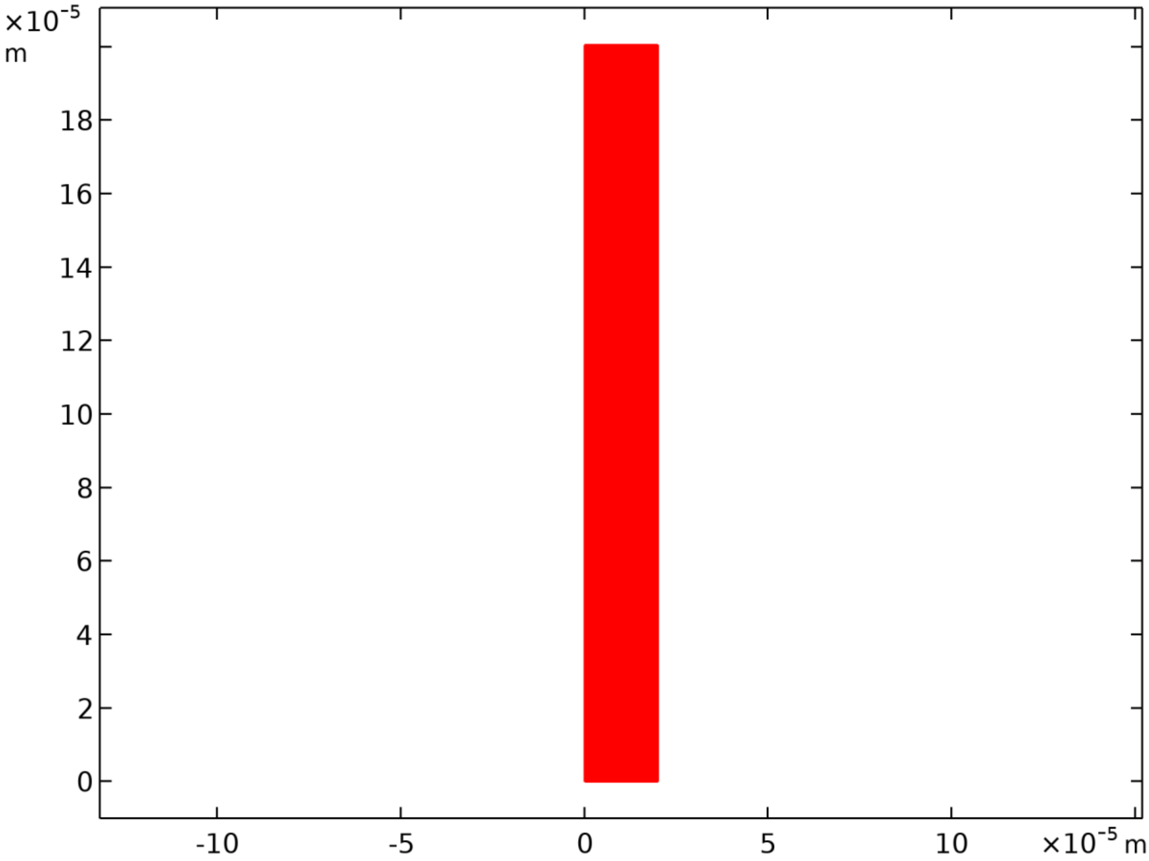

Dataset: Study 1/Parametric Solutions 1

4.2 PLOT GROUPS

4.2.1 Electrolyte Potential (tcd)

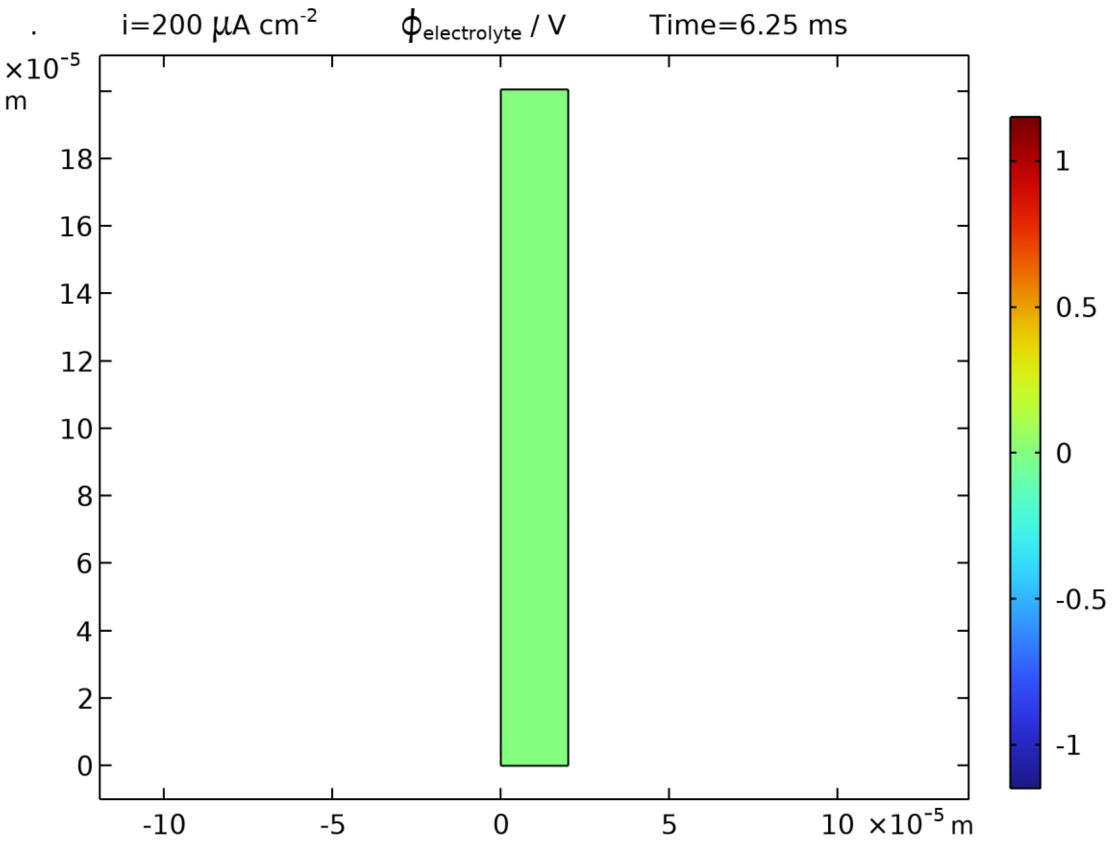

4.2.2 pH

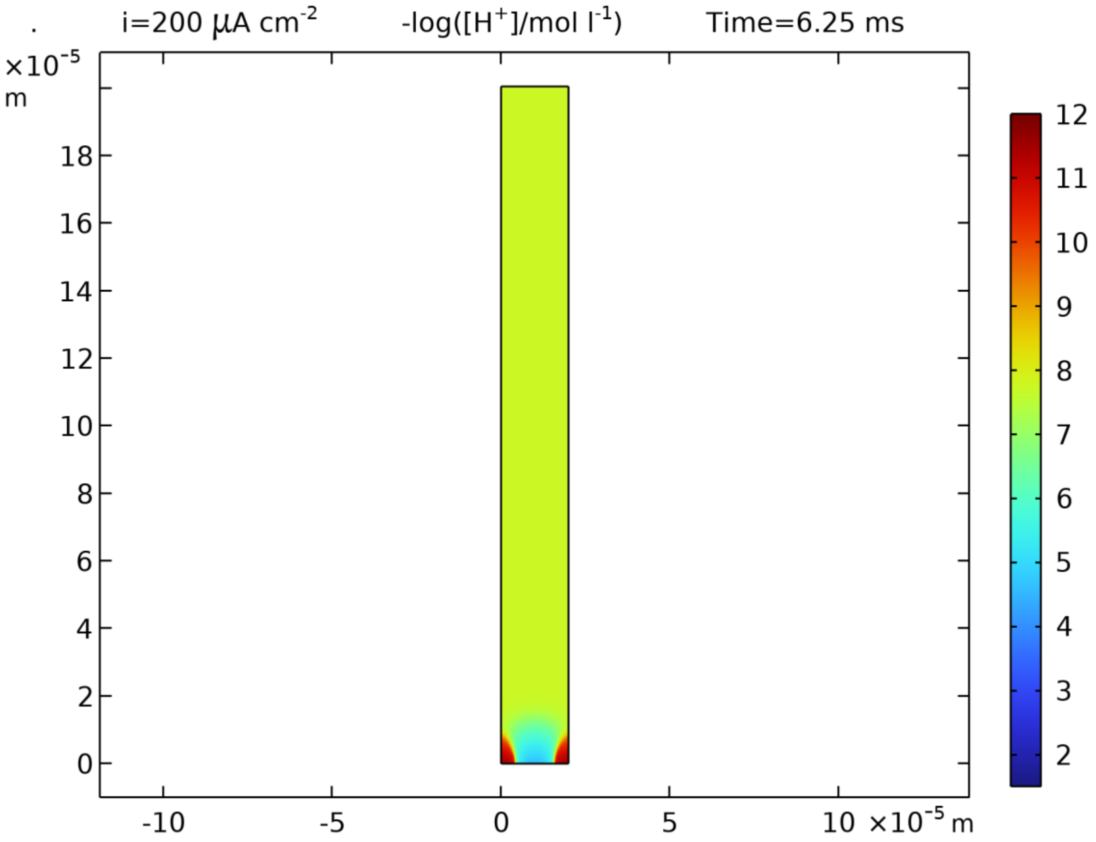

i=200 μA cm-2
-log([H+]/mol l-1)
Time=6.25 ms
×10-5 m
18
16
14
12
10
8
6
4
2
0
-10
-5
0
5
10 ×10-5 m
12
11
10
9
8
7
6
5
4
3
2

4.2.3 pAn

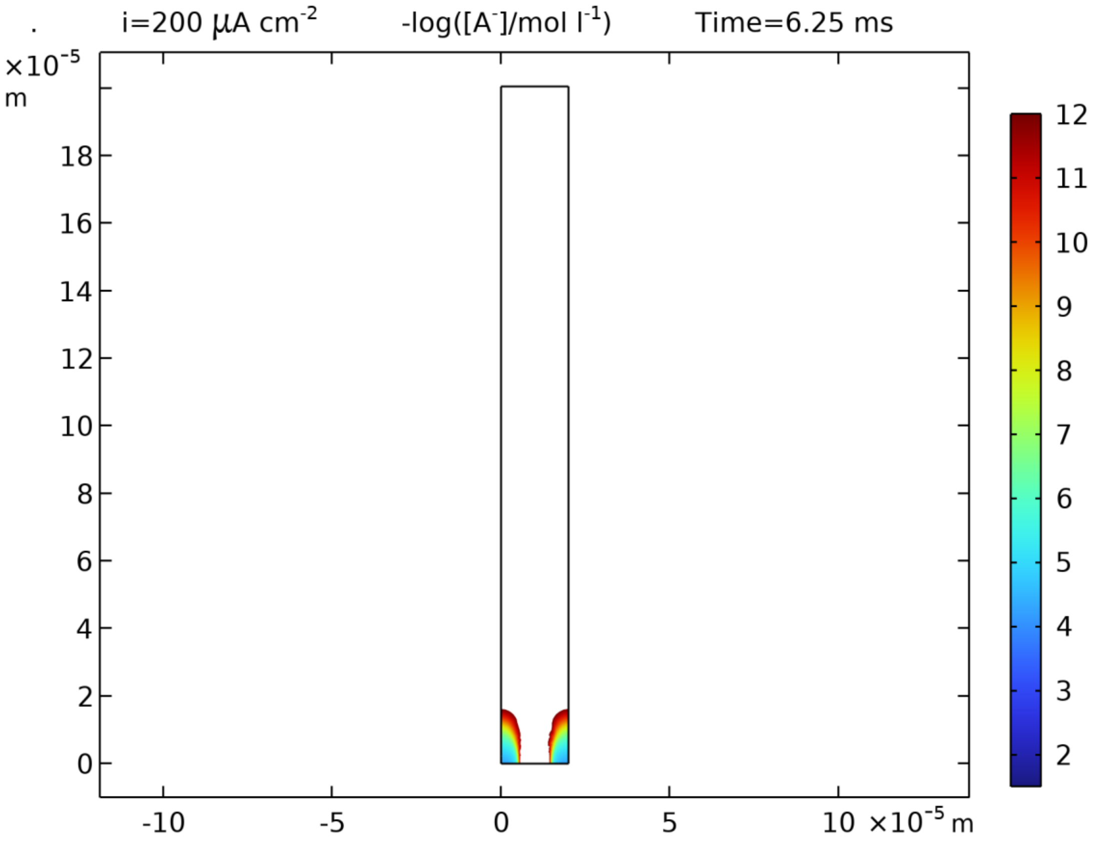

.
i=200 μA cm-2
-log([A-]/mol l-1)
Time=6.25 ms
×10-5 m
18
16
14
12
10
8
6
4
2
0
-10
-5
0
5
10 ×10-5 m
12
11
10
9
8
7
6
5
4
3
2

4.2.4 pBn

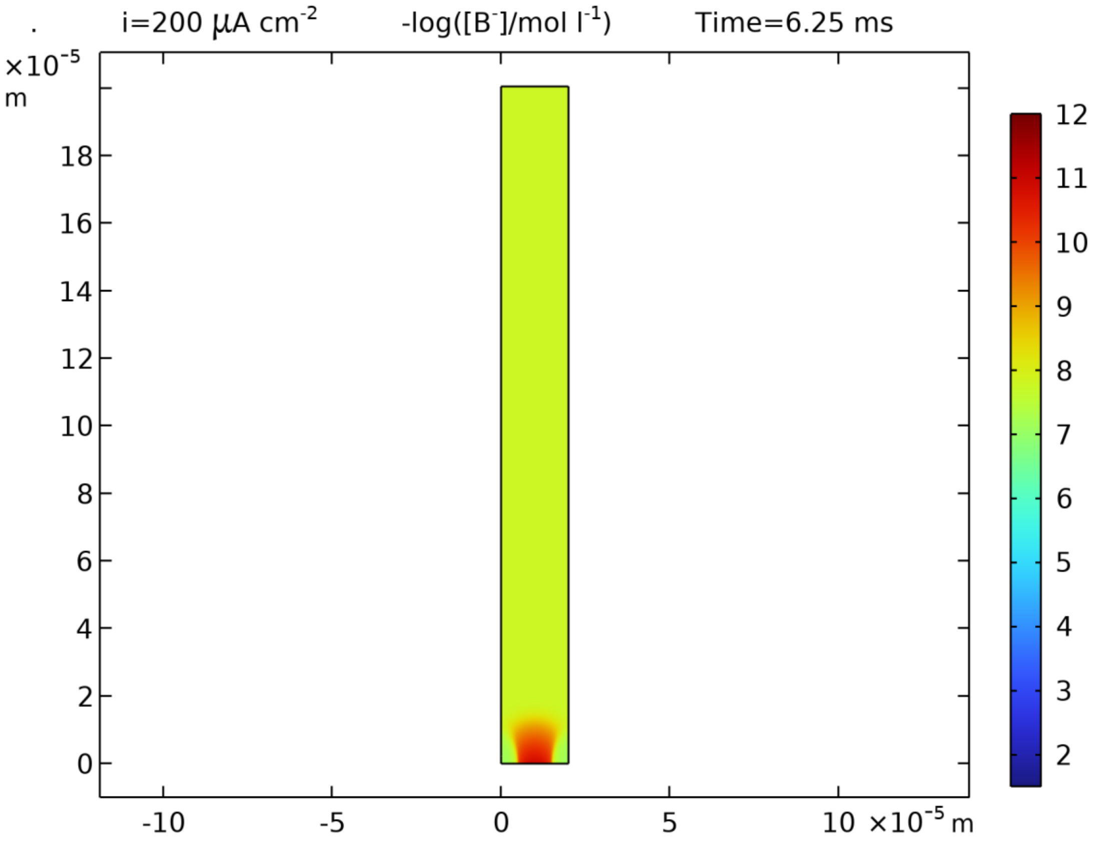

.
i=200 µA cm-2
-log([B-]/mol l-1)
Time=6.25 ms
×10-5 m
18
16
14
12
10
8
6
4
2
0
-10
-5
0
5
10 ×10-5 m
12
11
10
9
8
7
6
5
4
3
2

4.2.5 pAH

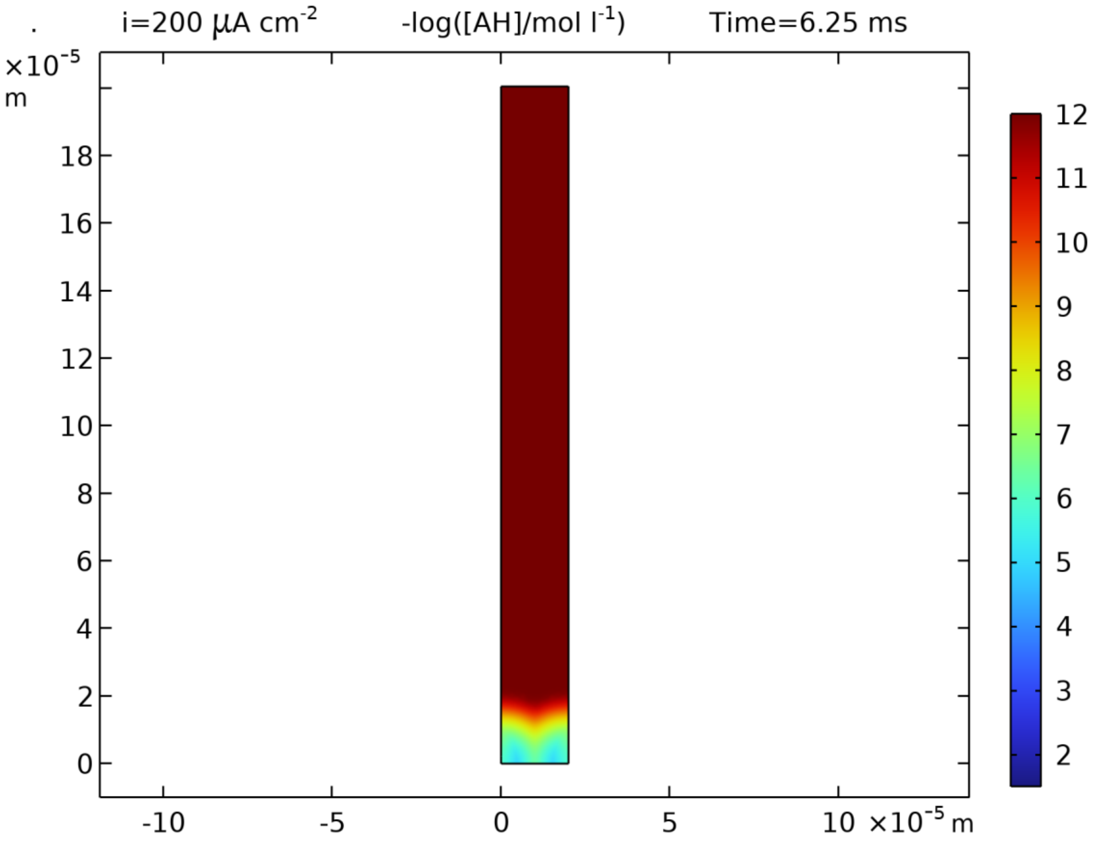

.
i=200 μA cm-2
-log([AH]/mol l-1)
Time=6.25 ms
×10-5 m
18
16
14
12
10
8
6
4
2
0
-10
-5
0
5
10 ×10-5 m
12
11
10
9
8
7
6
5
4
3
2

4.2.6 Average value of [AH] over space

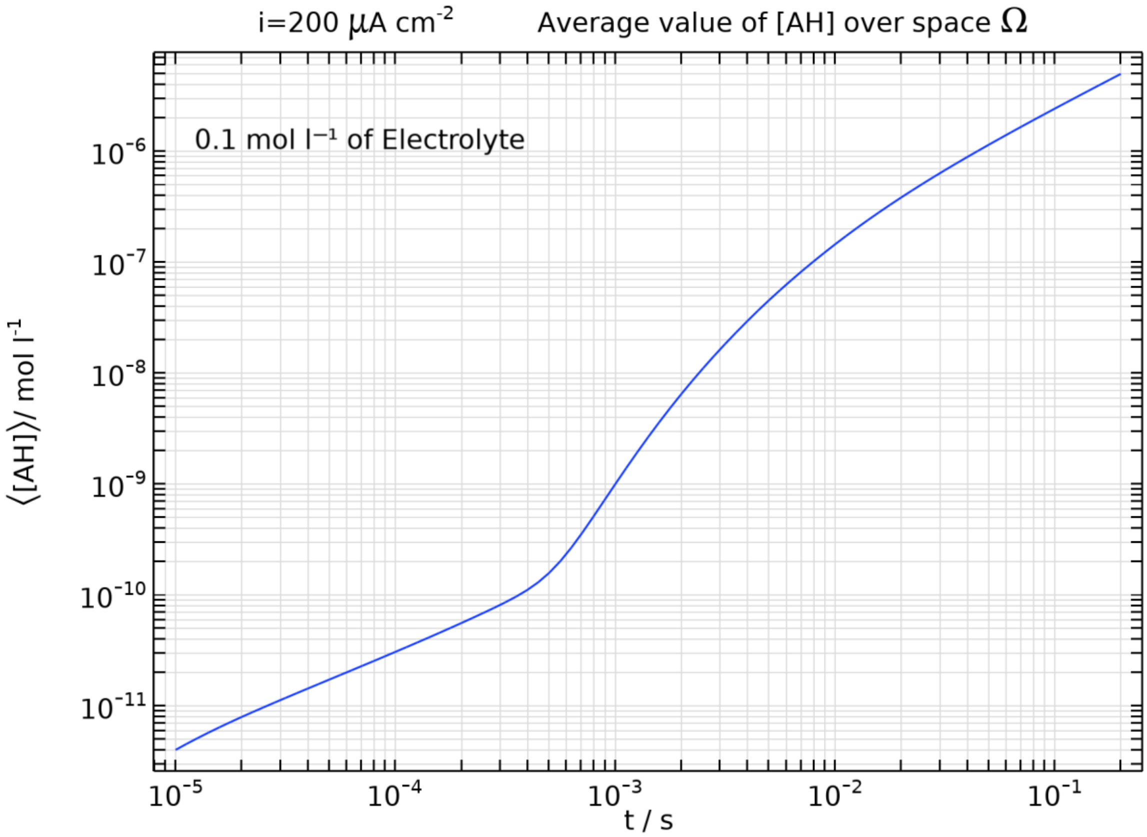

i=200 μ A cm^{-2} Average value of [AH] over space Ω

4.2.7 Average value of $[H^+]$ $[A^-]$ over space

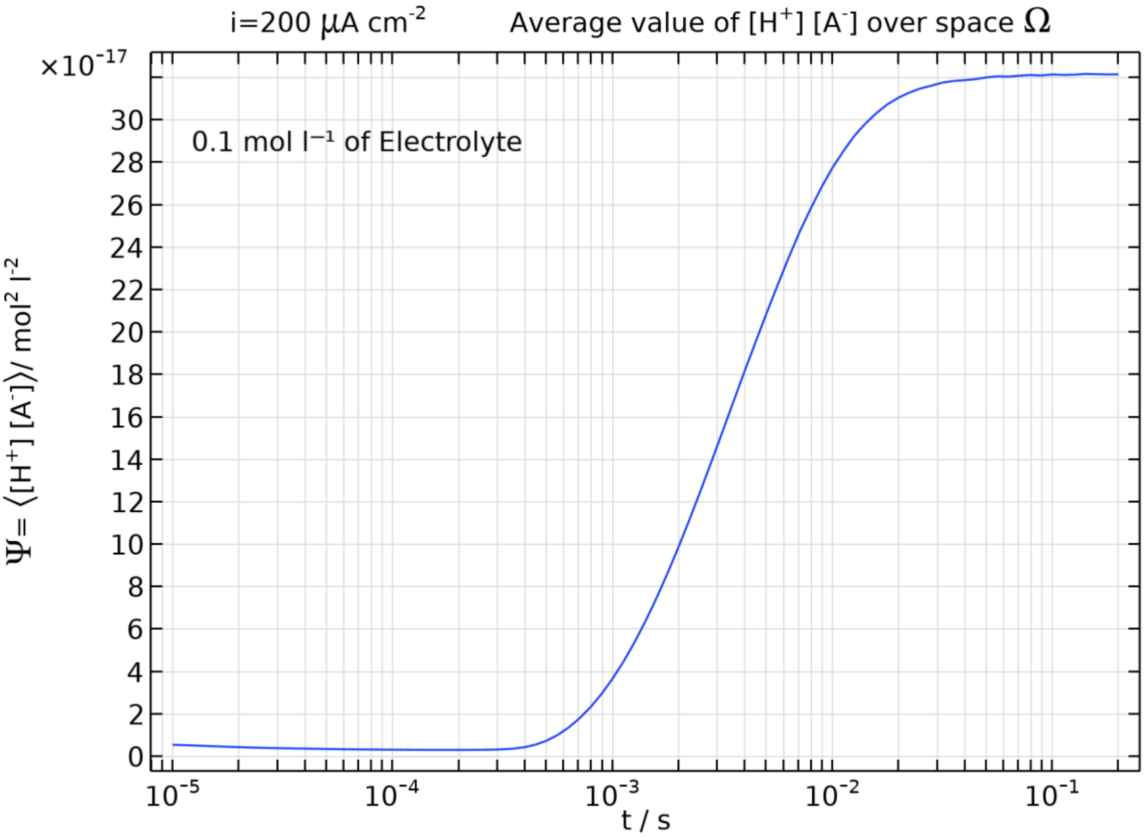

i=200 μ A cm^{-2} Average value of $[H^+]$ $[A^-]$ over space Ω
